# Study protocol: role of the blood-brain barrier in stress resilience: investigating new pathways towards Pharmacological augmentation of stress resilience (a PHASR-PP project study)

**DOI:** 10.1186/s40359-026-04118-z

**Published:** 2026-03-17

**Authors:** Frédérique MWM Maas, Sebastian Hachenberg, Julian Mituniewicz, Birgit Kleim, Susann Schweiger, Kenneth SL Yuen, Flurin Cathomas, Dorota Kobylińska, Oliver Tüscher, Raffael Kalisch

**Affiliations:** 1 Leibniz Institute for Resilience Research (LIR), Mainz, Germany; 2 Department of Adult Psychiatry and Psychotherapy, University Hospital of Psychiatry Zurich, University of Zurich, Zurich, Switzerland; 3 Department of Psychology, University of Warsaw, Warsaw, Poland; 4 Department of Psychology, University of Zurich, Zurich, Switzerland; 5 Institute of Human Genetics, Johannes Gutenberg University Medical Center, Mainz, Germany; 6 Neuroimaging Center (NIC), Focus Program Translational Neuroscience, Johannes Gutenberg University Medical Center, Mainz, Germany; 7 Department of Psychiatry and Psychotherapy, Johannes Gutenberg University Medical Center, Mainz, Germany; 8 Department of Psychiatry, Psychotherapy and Psychosomatic Medicine, University Medicine Halle (Saale) of the Martin Luther University Halle- Wittenberg (MLU) and German Center for Mental Health (DZPG), partner site Halle-Jena-Magdeburg, Halle, Germany

**Keywords:** BBB integrity, Stress resilience, At-risk, Prospective, Metformin, Placebo-controlled

## Abstract

**Background:**

Preclinical research indicates that the integrity of the blood-brain barrier (BBB) is an important resilience factor. Further, there is preclinical evidence suggesting that the mTOR pathway regulates BBB function and that pharmacological inhibition of this pathway may improve stress resilience.

**Methods:**

Translating these results to the human, we aim to establish a prospective association between BBB integrity and stress resilience in stressor-exposed individuals at risk to develop stress-related mental health problems. Using an observational study design, we primarily exploit assumed natural inter-individual variation in BBB integrity, assessed with neuroimaging, which we attempt to relate to inter-individual variation in stress resilience. We hypothesize that better whole-brain BBB integrity predicts better stress resilience. As a further measure to maximize sensitivity, we attempt to enhance inter-individual variation in BBB integrity by administering the indirect mTOR pathway inhibitor metformin to half of the participants, using a randomized parallel-group placebo-controlled double-blind multi-centre experimental design. This experimental-interventional approach also allows us to ask whether metformin improves whole-brain BBB integrity and whether this in turn improves stress resilience, via mediation analysis. Such a finding would suggest a causal role for BBB integrity in stress resilience.

In additional analyses, blood-based biomarkers and regional neuroimaging metrics are used as measures of BBB integrity, and the influence of socio-demographic, anamnestic, psycho-social, immunological, metabolic, and other neuroimaging variables on BBB function and stress resilience is also considered.

**Discussion:**

The study may, for the first time, delineate a relationship between BBB function and stress resilience in humans and potentially implicate BBB integrity as a causal factor in resilience. Findings may inform the development of novel pharmacological strategies to improve BBB function and stress resilience, to be investigated in follow-up studies.

**Trial registration:**

Registered under National Library of Medicine under the number NCT06965868 (https//clinicaltrials.gov/study/NCT06965868, initial registration date 04/24/2025, study start date 01/02/2026).

**Supplementary Information:**

The online version contains supplementary material available at 10.1186/s40359-026-04118-z.

## Background

### Rationale

#### Focus on resilience

Mental disorders are among the leading contributors to the global burden of disease [1], with depression and anxiety being the most outstanding [2]. These disorders can have significant personal, economic, and societal costs and are often triggered by stressor exposure and the ensuing stress reactions during childhood and adulthood [3]. Despite enormous efforts made in the last decades to understand stress-related pathophysiology and improve treatments, there has been no concomitant decline in disease prevalence. Insufficient investments into prevention research and prevention efforts (‘prevention gap’) have been identified as one major cause [4]. An approach focusing on the maintenance of mental health despite stressor exposure (i.e., stress resilience) is therefore an attractive complementary strategy to disease-oriented research to combat stress consequences, with the potential to prevent much individual suffering and societal burden [5]. This reasoning motivates research into the mechanisms and factors underlying stress resilience (resilience factors). Better understanding resilience may lead to new or improved prevention methods.

#### Focus on emerging adults

Stress-related mental disorders or problems often have their first onset during adolescence or early adulthood, where they tend to peak [6]. During the COVID-19 pandemic, youth and emerging adults were among the most strongly mentally affected groups [2, 7]. The vulnerability of this age group may partly relate to the critical transition many of them undergo from life in a familiar environment (family, school, friends) into the unfamiliarity and challenges of professional life or higher education, often accompanied by geographical relocation. This is supported by reports of frequent stress-related problems specifically in university student populations [8]. These data and the observation that early-onset stress-related problems are often associated with life-long mental vulnerability strongly suggest that investment in the mental health of emerging adults is likely to yield lasting gains and to be economically particularly efficient [6].

#### Focus on blood-brain barrier function, inflammation, and stress

The central nervous system has traditionally been viewed as an absolute immune-privileged site [9]. However, there is increasing evidence that there are ways by which the brain extensively interacts with other organ systems [10]. The blood-brain barrier (BBB) tightly controls the bidirectional communication between the central nervous system and the circulation and is therefore vital for brain protection and function. This complex selective interface – the neurovascular unit – consists of several specialized cell types, including non-fenestrated brain endothelial cells that are characterized by highly specific tight junctions sealing the para-cellular space, pericytes and smooth muscle cells that play a major role in controlling the cerebral blood flow, and astrocytic end-feet covering most of the vasculature [11]. The immune system and the BBB are tightly intertwined [11]. While under physiological conditions, most peripheral cytokines or immune cells cannot penetrate the BBB or depend on specialized transporters regulating their passage, pathological conditions such as acute or chronic inflammatory states can lead to increased BBB permeability [11, 12], involving the influx of potentially neurotoxic proteins or factors, such as peripheral IL-6 into the brain parenchyma [13]. Given that stress is associated with profound changes in the immune system [14], one could hypothesize that BBB integrity and function might also be impaired in stress-related conditions. A recent pre-clinical study showed that upon exposure to chronic social defeat (CSD), which is one of the best-validated murine stress models, stress-reactive (but not stress-resilient) mice showed increased BBB permeability. This was caused by a stress-induced downregulation of the endothelial tight junction gene/protein Claudin-5 resulting in the influx of proteins that can potentially alter neuronal function, e.g., peripheral IL-6 [15]. In the same study, the Claudin-5 gene was also shown to be downregulated in postmortem tissue from patients with major depressive disorder (MDD). The link between stress and BBB permeability was further substantiated in a study showing that hippocampal BBB permeability was increased in mice that underwent the learned helplessness paradigm and that BBB permeability and behavioral abnormalities could be reversed after pharmacologically blocking the cytokine TNFα [15]. Areas of the brain where BBB function appears to be particularly sensitive to stress include the hippocampus (HPC), the prefrontal cortex (PFC), and the ventral striatum (VS), which are also important nodes in brain networks regulating stress and emotion [13, 16].

#### Measurement of BBB function in humans

In humans, BBB permeability can be assessed using invasive methods, specifically contrast agent-enhanced magnetic resonance imaging (MRI) of the brain as a direct indicator [17] and measurement of plasma or cerebrospinal fluid (CSF) proteins as indirect indicators [18, 19]. Recently, a non-invasive MRI-based neuroimaging method without contrast agent has been developed [20, 21]. To date, however, the evidence linking BBB dysfunction and stress-related disorders exclusively stems from studies that have used indirect measures like vascular markers in circulation or ratios between CSF and blood proteins, such as the CSF/serum albumin quotient [22]. Because albumin is synthesized in the liver, not in the CSF, albumin measured in the CSF stems from the circulation and can be used as a proxy to assess blood-CSF leakiness. In a study performed on elderly women, those with MDD had a higher CSF/serum albumin ratio [23]. In addition, an early study showed increased levels of markers indicative of BBB permeability in MDD [24]. Another peripheral marker of BBB dysfunction is S100β. This calcium-binding protein, which is mainly expressed in glial cells, is normally not detectable in serum; however, it is elevated in the presence of BBB damage [25]. Several studies have reported increased levels of S100β in patients with MDD [26]. In addition, low-grade systemic inflammation (assessed by CRP, SAA, ICAM-1, IL-6, IL-8, TNF-α) and endothelial dysfunction (assessed by VCAM-1, E-selectin, VWF, ICAM-1) were both associated with depression, while endothelial dysfunction was further associated with chronicity of depressive symptoms [27, 28]. The anatomical localization of stress-related BBB leaks in the human brain cannot be established with these indirect marker methods, in contrast to neuroimaging methods.

In summary, in the context of a close interaction between peripheral inflammation and neurovascular dysfunction, there is increasing evidence that both contribute to the detrimental effects of stressor exposure on mental health, and improved neuroimaging methodology now allows for a more detailed and non-invasive investigation of the role of the human BBB in this interplay.

#### Stress-dependent regulation of behavioral and BBB function via the mTOR pathway

Recent animal work has identified the mTOR (mechanistic target of rapamycin) pathway as a likely mediator of the detrimental effects of exposure to a chronic social stressor on normal adaptive behavior in mice [29]. Specifically, pathway analyses of differentially expressed genes after CSD in hippocampal cells indicated mTOR pathway upregulation in stressor-reactive mice, whereas mice that maintained behavioral functioning despite CSD (stress-resilient mice) showed relatively reduced expression of downstream mTOR effectors and enhanced expression of negative mTOR regulators (MAPK and PI3K-Akt pathways). Importantly, administration of the mTOR inhibitor (and MAPK and PI3K-Akt upregulator) rapamycin [30] over several days following CSD prevented CSD-induced behavioral impairments, that is, made animals more stress resilient [29].

Interestingly, mTOR-related differential gene expression was observed not in neuronal, but mainly in glial, mural, and endothelial cells, pointing towards a role for the BBB in stress vulnerability vs. resilience. The mTOR signalling pathway is an evolutionarily conserved pathway that senses and integrates a broad range of environmental cues, e.g., growth factors or immune modulators, to regulate several homeostatic processes, including in the vasculature [31]. In various mouse models of neurological and inflammatory diseases that have been associated with impairments in the neurovascular unit and reduced BBB integrity, rapamycin had a positive effect on BBB and vascular function [32–35]. An additional BBB-protective effect of rapamycin beyond mTOR inhibition may be achieved by its anti-inflammatory actions [36].

Taken together, pharmacological mTOR pathway inhibition and immunosuppression, as effectuated for instance by rapamycin, presumably enhance resilience via promotion of BBB integrity and reduced exposure of the brain to mediators of inflammation.

#### Metformin as an experimental tool to enhance BBB function in humans

These combined insights from human and animal studies give rise to the hypothesis that better BBB integrity in stressor-exposed humans reduces their risk to develop stress-related mental health problems, that is, BBB integrity in the face of stress is an inter-individual resilience factor. Further, next to naturally occurring (spontaneous) inter-individual variability in BBB integrity, inter-individual differences may also be generated pharmacologically via mTOR pathway inhibition (relative to a control pharmacological intervention). Improvement of BBB integrity as a result of mTOR pathway inhibition should enhance resilience (mediation). Therefore, an experimental design where natural and pharmacologically induced inter-individual variance in BBB function is combined should be highly sensitive to demonstrate effects of BBB integrity on stress resilience.

To pharmacologically manipulate BBB function, rather than using the mTOR inhibitor and immunosuppressant rapamycin, it is likely advantageous to use the indirect mTOR inhibitor metformin. Rapamycin has already been employed in humans (as sirolimus), however, with an unfavorable side effects profile. Metformin inhibits mTOR via PI3K-Akt [37–39]. Orally administered metformin has been used for decades in millions of patients in the prevention and treatment of type 2 diabetes and has recently been suggested to have anti-aging and anticancer effects, among others [37–39]. Metformin has a highly favorable safety profile and can be administered over extended times; adverse effects can be avoided by excluding individuals with kidney dysfunction, liver disease, or vitamin B12 deficiency or in pregnancy and by gradual dosing [37, 38]. The possibility to administer metformin over longer time periods is important since the beneficial effect on resilience (and presumably BBB function) of rapamycin in stressed mice was achieved through chronic treatment [29]. Metformin is off-patent and relatively inexpensive.

Importantly, there are indications that metformin also has anti-inflammatory effects [39, 40] and may protect against cardiovascular disease [37–39], and recent work in mice has shown that metformin attenuates BBB disruption following middle cerebral artery occlusion [41], suggesting that the protection of BBB function is a plausible working mechanism of metformin.

### Aims and objectives

#### Aims

The overarching aim of the study is to better understand the relationship between BBB function and stress resilience and to investigate a potential causal role for BBB function in stress resilience in humans.

**Fig. 1:**
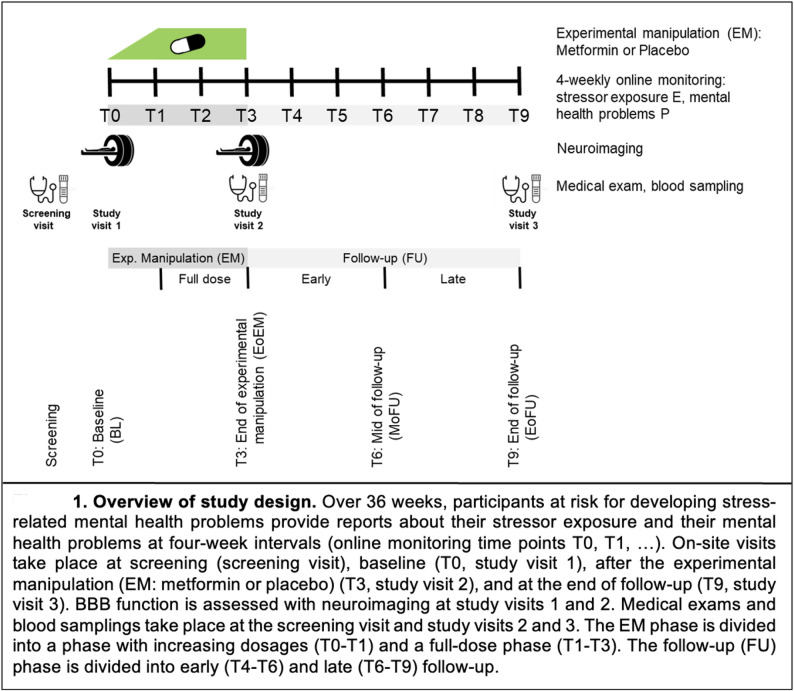
Overview of study design. Over 36 weeks, participants at risk for developing stress-related mental health problems provide reports about their stressor exposure and their mental health problems at four-week intervals (online monitoring time points T0, T1, …). On-site visits take place at screening (screening visit), baseline (T0, study visit 1), after the experimental manipulation (EM: metformin or placebo) (T3, study visit 2), and at the end of follow-up (T9, study visit 3). BBB function is assessed with neuroimaging at study visits 1 and 2. Medical exams and blood samplings take place at the screening visit and study visits 2 and 3. The EM phase is divided into a phase with increasing dosages (T0-T1) and a full-dose phase (T1-T3). The follow-up (FU) phase is divided into early (T4-T6) and late (T6-T9) follow-up

Specifically, the study aims to establish a prospective association between BBB integrity and stress resilience in stressor-exposed individuals at risk to develop stress-related mental health problems. It primarily builds on assumed natural inter-individual variation in BBB integrity, assessed with neuroimaging, which it attempts to relate to inter-individual variation in stress resilience in an observational approach. It is hypothesized that better BBB integrity predicts better stress resilience. Because the animal literature does not allow us to estimate the duration of the hypothesized effect, both a short-term and a long-term resilience outcome are employed. As a further measure to maximize sensitivity, we attempt to enhance inter-individual variation in BBB integrity in the study sample by administering the indirect mTOR pathway inhibitor metformin to half of the participants, using a randomized parallel-group placebo-controlled double-blind multi-center multimodal pre-registered experimental design. This experimental-interventional approach allows us to ask whether metformin improves BBB integrity and whether this in turn improves stress resilience (mediation). For an overview of the study design, see Fig. 1.

### Objectives

To achieve our goal, we have defined several objectives within the study. The **primary objective O1** consists of three sub-objectives. The first **sub-objective O1a** is to test whether better BBB integrity, assessed with neuroimaging at the whole-brain level, is prospectively associated with better short-term resilience (i.e., reduced average stressor reactivity at time points T4–T6 during early follow-up; comp. Figure 1). The second **sub-objective O1b** is to test whether an experimental pharmacological manipulation — the administration of metformin from time points T0 to T3 — improves BBB integrity at time point T3 (i.e., at the end of the experimental manipulation), relative to baseline (T0). The third **sub-objective O1c** is to test whether the effect of the experimental manipulation on BBB integrity at T3 mediates a potential effect of the manipulation on short-term resilience (i.e., reduced average stressor reactivity at T4–T6).

The **secondary objective O2** consists of two sub-objectives. The first **sub-objective O2a** is to test whether better BBB integrity, assessed with neuroimaging at the whole-brain level, is prospectively associated with better long-term resilience (i.e., reduced average stressor reactivity at time points T4–T9 during the whole follow-up period). The second **sub-objective O2b** is to test whether the effect of the experimental manipulation on BBB integrity at T3 mediates a potential effect of the manipulation on long-term resilience (i.e., reduced average stressor reactivity at T4–T9).

In additional exploratory analyses, blood-based biomarkers are used as measures of BBB integrity, and the influence of socio-demographic, anamnestic, psycho-social, immunological, and metabolic covariates on BBB function and stress resilience is also considered. We will also explore regional neuroimaging measures (esp. of hippocampus, ventral striatum, and prefrontal cortex) of BBB integrity as potential predictors of stress resilience and mediators of the experimental manipulation effects on stress resilience.

## Methods

### Design

**Fig. 2:**
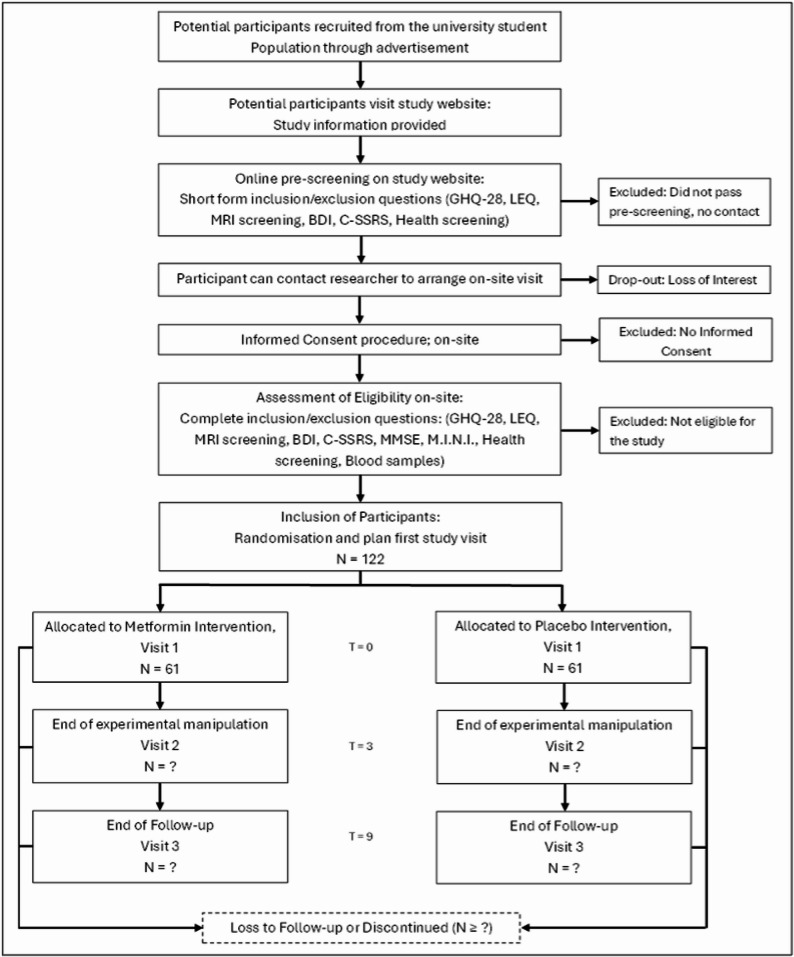
Participant timeline

The study is a prospective, randomized, parallel-group, placebo-controlled, double-blind, multi-center experimental-interventional trial conducted in participants at risk for stress-related mental health problems in order to investigate the relationship between individual variation in BBB integrity and individual variation in stress resilience, making use of a 12-week treatment with orally administered metformin (850 mg twice daily at full dose) as an experimental tool to enhance individual variation in BBB integrity. The duration of the study for a participant will be 36 weeks in total. The attempted sample size is *N* = 109. See Fig. 1 for an overview of the study design which is described below and see Fig.  2 for the particpant timeline.

Before the on-site screening visit, an anonymous online pre-screening will be done via the online platform Sosci-survey [42].

During the on-site screening visit, informed consent will be obtained from potential participants. Then, they will be assessed for eligibility, including a medical exam, blood sampling, and a psychological exam.

Subsequently, included participants provide reports about their stressor exposure and potential mental health problems, as part of a regular four-weekly online monitoring (time points T0 to T9).

At time point T0 (baseline, week 1), a first study visit is conducted on site, including neuroimaging, randomization, and distribution of the study drug and the participant diary. Participants also fill in an online questionnaire battery.

The experimental manipulation (EM) phase spans time points T0 to T3 and consists of four weeks of dose increase (T0 to T1; first and second weeks: 500 mg once daily, third and fourth weeks: 500 mg twice daily) and eight weeks of full-dose regimen (T1 to T3; 850 mg twice daily). The study drug is self-administered.

A second on-site study visit at time point T3 (end of EM, week 12) consists of a medical exam, blood sampling, neuroimaging, rendition of the study drug blisters and the participant diary, and adverse event (AE) monitoring. Another online questionnaire battery is administered.

The follow-up phase (time points T4 to T9) is separated into early (T4 to T6) and late (T7 to T9) follow-up and followed by a third on-site study visit (end of follow-up, week 36) with a medical exam, blood sampling, and AE monitoring. The online questionnaire battery is administered a last time.

### Setting

The study will be conducted at three different study sites (Mainz, Germany; Zurich, Switzerland; Warsaw, Poland). The setting is urban. Participants are university students between 18 and 25 years of age.

### Recruitment

We distribute participant recruitment across three different countries, since this will (a) increase the heterogeneity of the study sample compared to a study in only one country (and, consequentially, increase the likelihood that the results are generalizable) and (b) enhance chances to recruit a sufficient number of participants in the planned recruitment period of ten months. The recruiting centers are all part of the former DynaMORE consortium (www.dynamore-project.eu**)** and have proven to be able to recruit sufficient numbers of participants from the target population (university students) in a variety of studies. We plan to recruit at least 45% of participants of either male or female gender, leaving the remaining 10% to any gender, while explicitly also addressing non-binary persons in the recruitment campaign.

### Eligibility criteria

Inclusion criteria (participants meeting all of the following criteria will be considered for enrollment in the study):

1. Absence of mental disorder diagnosis.
2. University students.
3. General Health Questionnaire (GHQ-28) ≥ 20.
4. Three or more adverse life events acc. to life-events questionnaire (LEQ) in the past.
5. Beck Depression Inventory (BDI) ≤ 14 & Columbia-Suicide Severity Rating Scale (C-SSRS) ≤ 1. Thereby concurrent depression and suicidality are excluded.
6. Age 18 to 25 years.
7. Ability of participant to understand character and individual consequences of the study (Mini Mental State Exam (MMSE) Folstein version > 28).
8. Signed and dated informed consent of participant.

Exclusion criteria (participants presenting one of the following criteria will not be enrolled in the study):

9. Life-time and current diagnosis of any severe mental disorder determined by M.I.N.I. diagnostic interview.
10. Known history of brain injuries or neurodevelopmental disorder.
11. Evidence of neurodegenerative disorder (e.g., Parkinson).
12. Multimorbidity or significant organ (esp. liver or renal) dysfunction or manifest diabetes (determined by HBA1C levels in blood sample) or substance abuse (esp. alcohol).
13. Contraindication to metformin such as renal insufficiency (Creatinin-Clearance< 60 ml/min), recent (< 3 month) ischemic events (e.g. myocardial infarction or stroke).
14. Women of childbearing age, who do not practice a medically accepted contraception (i.e., systematic contraceptives, diaphragm, condoms with spermicide, sexual abstinence) during the study and during a 2-years post-study period and who do not present a negative pregnancy test (serum or urine).
15. History of hypersensitivity to the study drug, to any drug with similar chemical structure, or to any excipient present in the pharmaceutical form of the study drug.
16. Diabetes type 2 (would result in interference with the experimental manipulation), determined by HBA1C levels in blood sample.
17. Participation in other studies employing a drug during the present study or within the last three months.
18. Current use of antidiabetic, weight-loss, or psychoactive medication or substances.
19. Pacemaker, implanted medical pumps, implanted cardiac catheters or acute or unstable heart disease (angina pectoris).
20. Intracranial implant (aneurysm clips, shunts, stimulators, cochlear implants or electrodes) or other metallic objects inside or near the head (mouth excluded) that cannot be removed.
21. Claustrophobia or another contraindication to MRI.
22. Insufficient language skills (required language skills: German in Germany and Switzerland, Polish in Poland).

### Informed consent process

Before being admitted to the study, the participant must consent to participate after being fully informed about the nature, scope, and possible consequences of the study. After reading the informed consent document, the participant must give consent in writing. The participant's consent must be confirmed by the personally dated signature of the participant and by the personally dated signature of the person conducting the informed consent discussions.

## Allocation

Participants will be randomly assigned to either experimental or control group with a 1:1 allocation as per a computer-generated, block-wise randomization schedule, separately per site. A randomization list will be generated centrally at the Mainz research site. One copy of the randomization list will be sent to the pharmacy of the other respective sites. At the randomization visit (study visit 1 at T0), each participant eligible for study participation will receive the next consecutive randomization number from a block of randomization numbers per site. The randomization list will be kept in safe and confidential custody at each study center site.

### Blinding (masking)

Experimental manipulation groups are completely masked (double-blind study). In addition to the study drug, the investigator will receive a set of sealed envelopes, one for each randomization number. These envelopes contain information on each participant’s study drug and are to be opened only under circumstances in which it is medically imperative for diagnostic or therapeutic decisions to know what the participant is receiving. The investigator must report all code breaks (with reason) as they occur on the corresponding CRF.

### Adherence and remuneration scheme

To promote adherence, a step-wise reimbursement scheme is implemented (here described in euros but will be paid in the equivalent in the local currency per study site). Participants receive a first reimbursement of max. 40 € after the screening visit (30 € for participation in the medical exam and online questionnaires, 10 € specifically for blood draws), max. 50 € after study visit 1 (30 € for neuroimaging, 20 € for the online questionnaires, max. 290 € after study visit 2 (50 € for medical exam and online questionnaires, 10 € for blood draws, 30 € for neuroimaging, 200 € for study drug administration, where 10% missed administrations are allowed), and max. 240 € after the study visit 3 (50 € for medical exam and online questionnaires, 10 € for blood draws, 180 € for online monitoring of stressor and internalizing symptoms, the latter proportional to completion). The total possible reimbursement is 620 € in Germany, thus a total of 700 CHF in Switzerland and 1900 PLN in Poland.

### Rules for discontinuation

Discontinuation criteria are:

- A participant may discontinue participation due to any of the following reasons:
  at their own request or at request of the legal representative
  any medical condition demanding the immediate discontinuation of (potential) metformin treatment, e.g., need for urgent anesthesia, renal failure etc.
  for safety reasons at the request of the investigator or request of a regulatory agency
  significant AEs related to the experimental manipulation (participants will be followed up for manipulation response and safety)
  participant is non-compliant or not sufficiently compliant with the study procedures/study protocol
  if, in the investigator’s opinion, continuation of the study would be detrimental to the participant’s well-being.
  participant needs medication not allowed in the protocol during the study
  any clinically significant change in participant’s pre-study medical condition
- For the following reasons, a study site may be closed at the discretion of the investigator:
  medical or ethical reasons that are detrimental to the continued performance of the study
  difficulties in the recruitment of participants (i.e., which fail to include at least a third of estimated participants to be included by mid of the recruitment period (6.5 months after last participant-in))
  critical protocol violations
  violations of legal and ethical regulations
  non-compliance of the study site investigators
- The whole study may be discontinued at the discretion of the investigator for the following reasons:
  new risks for participants become known
  occurrence of AEs unknown to date in respect of their nature, severity, and duration or the unexpected increase in the incidence of known AEs
  medical or ethical reasons that are detrimental to the continued performance of the study
  difficulties in the recruitment of participants

#### Concomitant care (emergency procedures)

In this study, no patients will be enrolled, and no concomitant care will be provided. In cases of emergency during and following a participant’s participation in the study, the investigator will ensure that adequate medical care is provided to a participant for any AEs. If it is medically imperative to know what study drug the participant is receiving or has received, the investigator or an authorized person will break the blind.

### Interventions

#### Experimental manipulation

The intervention is an experimental manipulation (EM) using metformin compared to placebo. Metformin is used off-label. Since the EM is not employed to test or achieve a clinical effect, we consider placebo comparison ethically acceptable. Eligible participants will be randomized in equal proportions to the experimental (metformin) and control (placebo) groups. Metformin and placebo are self-administered orally with meals in the form of identically looking capsules following an identical administration scheme. Participants in the experimental group will take 500 mg metformin once daily during the first and second weeks of the EM phase of the study, then 500 mg metformin twice daily during the third and fourth weeks of the EM phase, and finally 850 mg twice daily (full dose) during the fifth to 12th week of the EM phase. Capsules will be provided by the pharmacy of the University Medical Center Mainz.

#### Rationale for choice of dose, mode, and scheme of intervention

We have no specific information available on the optimal dose of metformin to achieve BBB effects. We therefore rely on experiences with the use of metformin for current indications (esp. diabetes), where dosages range from 250 to 3000 mg/day over months or years [3, 43]. 850 mg twice daily, administered daily, is an average dose used for the treatment of Diabetes mellitus type 2. In a comparable setting (safety and tolerability of metformin for treatment of amnestic mild cognitive impairment), most participants tolerated dosages between 1000 mg and 1500 mg a day [23]. Similar regimens are used in trials of metformin in aging-related diseases. These data define a safe range, which to exploit enhances chances of finding an effect (sensitivity criterion). At the same time, it needs to be considered that our study participants are formally mentally healthy and that the motivation to participate and the adherence during the study may be negatively affected by a high dose (feasibility criterion). Minimization of participant burden is a further relevant aspect (safety criterion). In this trade-off, we have chosen a daily oral dose of 1700 mg (850 mg BID with meals) during the full-dose administration (weeks 5 to 12) that is still well in the range of current dosage regimens and also well below the maximum recommended dose of 3000 mg, and we have decided to restrict administration to three months overall. We further opt for an initial titration starting comparably low at 500 mg daily the first two weeks, followed by 1000 mg daily during the third and fourth weeks.

### Data collection

#### Outcome stress resilience

Stress resilience is quantified as inverse stressor reactivity (that is, reactivity of participants’ mental health to their stressor exposure). Following study visit 1, a schedule with the participant’s dates for all four-weekly online monitoring time points (T0 to T9) will be uploaded to SoSci Survey [42] to enable automatic e-mail dispatch. At each time point, participants have two days to fill in online questions about mental health (Patient Health Questionnaire - Anxiety and Depression Scale, (PHQ-ADS)) and stressor exposure (Mainz Inventory of Microstressors (MIMIS), life-events questionnaire (LEQ)) (see appendix 2).

#### Outcome whole-brain BBB integrity

Whole-brain BBB integrity is quantified as inverse whole-brain water exchange rate (kw) from blood to brain tissue (BBB permeability) and will be assessed with neuroimaging. Data will be collected at study visits 1 and 2 (T0, T3). The personnel at each study site will be trained during pre-study site visits in the study requirements.

Brain imaging data will be acquired on 3 T MAGNETOM PRISMA systems (Siemens Healthineers, Erlangen, Germany) with 32-channel head coils. To assess BBB permeability, a multi-TE, Hadamard pseudo-continuous arterial spin labelling (ME-pcASL) [44] with 3D GRASE readout (TR=4500ms, TE=17.30ms, 51.90ms, 86.50ms, 121.10ms, 155.70ms, 190.30ms, 224.90ms, 259.60ms, sub-bolus duration=1000ms, PLD=600ms, aqcuired with a Hadamard-4 matrix, FOV=320mm, FOV phase=50%, turbo factor=2, EPI factor=16, bandwidth=2440Hz/Px, GRAPPA=2, voxel size=2.5x2.5x5mm) will be used. As a measure to assure data quality and assess robustness of results in additional analyses, a second ASL sequence (diffusion prepared pseudo-continuous ASL, DP-pcASL) [45] with 3D GRASE readout (TR=4100ms, TE=31.1ms, label/control duration=1500ms, PLD=500/1000/1500/2000/2500ms, FOV=240mm, turbo factor=14, EPI factor=64, bandwidth=2604Hz/Px, voxel size=2.5x2.5x3mm) will be used.

#### Retention

Participants may withdraw from the study for any reason at any time. The investigator also may withdraw participants from the study for any of the reasons detailed in the stopping rules (see ‘Rules for discontinuation’ on p. 9). Once a participant is enrolled or randomized, the study site will make every reasonable effort to follow the participant for the entire study period. It is projected that the rate of attrition will be at most 10%.

#### Other data

Socio-demographic and anamnestic information (see appendix 1) will be collected during online pre-screening and at the screening visit. Blood-based molecular and cellular markers of immune, metabolic, and BBB function (including proteins, peptides, RNAs, metabolites) will be collected at the screening visit and study visits 2 and 3 (T3, T9). During study visits, participants fill in an online battery of psycho-social questionnaires (appendix 2). Additional questions on sleep quality, mood, and menstrual cycle status are included in the online monitoring questionnaire (appendix 2). Additional neuroimaging measures will be collected at the study visits 1 and 2 (T0, T3). These include resting-state functional MRI and anatomical MRI (T1, T2) scans (appendix 3).

### Data monitoring

Monitoring will be done by personal visits from study-external monitors. Pre-study visits will be conducted, and the reports will be reviewed by the investigator. To initiate the study, the monitor will visit the local study site and study centers. The monitor shall ensure that the investigator and their staff understand all requirements of the protocol and their regulatory responsibilities. The monitor will ensure that the investigator will maintain a list of sub-investigators and other appropriately qualified persons to whom he or she has delegated significant study-related duties (personnel log). Each site will be visited by the monitor at regular intervals to ensure compliance with the study protocol, GCP, and legal aspects. The monitor will review the entries into the case report forms (CRFs) for completeness and correctness and verify the entries on the basis of the source documents. The presence of correct informed consents will be checked for every participant. Details will be specified in the monitoring manual for this study. The investigator must allow the monitor to look at all relevant documents and must provide support at all times to the monitor. By frequent communications (letters, telephone, fax), the monitor will ensure that the study is conducted according to the protocol and regulatory requirements.

### Safety/harms

An adverse event is defined according to GCP as any untoward medical occurrence in a participant treated with a pharmaceutical product and which does not necessarily have a causal relationship with this treatment. An AE can therefore be any unfavorable and unintended sign (including an abnormal laboratory finding), symptom, or disease temporally associated with the use of a study drug, whether or not related to that drug. 

Participants will be carefully monitored for AEs by the investigator at each study visit. The intensity of the AEs and the causal relation to the study drug and/or procedures will be assessed. The period of observation for collection of AEs extends from the time the participant has signed the informed consent document up to the end of the 24 weeks follow-up period. If the investigator detects a serious AE in a study participant after the end of the period of observation and considers the event possibly related to the prior study, they are instructed to contact the Data and Safety Monitoring Board (DSMB) to determine how the AE should be documented and reported. All AEs reported by the participant or detected by the investigator will be documented on the appropriate pages of the CRF. All participants who have AEs, whether considered associated with the use of the study drug or not, will be monitored to determine the outcome.

Serious AEs (SAEs) will immediately (within 24 hours of the investigator’s awareness) be reported to the DSMB. The initial SAE Report will be as complete as possible, including the essential details of participant’s identification (screening number, random number), the SAE (medical term, diagnosis), the study drug, and the assessment of the causal relationship between the event and the study drug. The SAE report will be reviewed and signed by the investigator. The investigator will provide related additional information on the clinical course and the outcome of each SAE as soon as possible (Follow-up report). The “Serious Adverse Event Form” is provided in the investigator site file (ISF). The investigator is also instructed to inform the study monitor in all cases.

Any pregnancy diagnosed in a female participant or in the female partner of a male participant during the experimental manipulation will be reported immediately using the “Pregnancy Reporting Form” (provided in ISF) to the DSMB. Although no teratogenic effects of metformin are known or expected, an extended contraception requirement is implemented as a precautionary measure to safeguard embryonic and fetal development. These precautions are necessary to address potential, although theoretical, risks and to comply with regulatory and ethical obligations. While direct exposure of the participant´s partner to metformin does not occur, the possibility of drug residues being prominent in semen and thus posing a theoretical risk can never entirely be excluded. Therefore, prompt reporting of a partner´s pregnancy allows for appropriate risk evaluation and documentation.

The investigator will ensure that all legal reporting requirements are met. The investigator is responsible for the continuous safety evaluation of the study drug and the study. The investigator will conduct the management of SAEs and the expedited reporting as required by GCP regulation (GCP-V). Suspected unexpected serious adverse reactions (SUSARs) and safety issues as defined by GCP-V are determined for expedited reporting. The ethics committees should be notified as soon as possible but not later than 15 calendar days if the event is non-fatal and 7 calendar days if it was fatal. All co-investigators and sub-investigators will be informed, too. Workflow and procedures concerning SAE management will be described in a separate document (e.g., Safety manual). During the study, the investigator will submit the annual safety report including a list of all serious adverse reactions to the ethics committee(s) once a year.

All observations pertinent to the safety of the study medication will be recorded on the CRF and included in the final report. Additionally, to the registration of the AEs and serious AEs, we will conduct further assessments of safety at every visit.

Relevant additional illnesses present at the time of informed consent are regarded as concomitant illnesses and will be documented on the appropriate pages of the CRF.

Relevant treatments administered to the participants on entry to the study or at any time during the study are regarded as concomitant treatments and will be documented on the appropriate pages of the CRF.

## Outcomes

### Quantification of stress resilience

Resilience is quantified using a stressor reactivity (SR) score [46]. SR is the individual deviation from the normative relation between stressor exposure (E) and mental health problems (P) in the study sample. A positive SR score indicates more mental health problems than expected based on the normative E-P relationship in the study sample. A negative SR score indicates less mental health problems than expected. Hence, SR is an inverse continuous expression of an individual’s stress resilience. 

Specifically in this study, SR is calculated as an individual's residual from a regression of mental health problems P [z-standardized sum score of anxiety and depression symptoms, as assessed with the Patient Health Questionnaire - Anxiety and Depression Scale (PHQ-ADS; see appendix 2) during online monitoring] on stressor exposure E [z-scored sum of occurrences of all daily hassles (DHs), as assessed with the Mainz Inventory of Microstressors (MIMIS; see appendix 2) during online monitoring] in the study sample.

To build the SR score, first, E is calculated for every four-weekly monitoring time point after T0 (T1 to T9 in Figure 1) as the z-scored sum of occurrences of all DHs. Life event occurrence (z-scored sum of occurrences of all life events, assessed with a life-events questionnaire (LEQ; see appendix 2)) at the same monitoring time points, is expected to be less frequent than DH occurrence but, if reported, expected to correlate with DH occurrence. (This will be used to corroborate the validity of the DH-based E score. If we find that adding LE occurrence to DH occurrence (by averaging the two z-standardized occurrence scores in a given study phase) explains significantly more variance in P in that study phase than the DH occurrence alone, then we will use the average occurrence score to calculate E for that study phase). For each monitoring time point, P is also calculated as the z-standardized sum score of anxiety and depression symptoms, as assessed with the PHQ-ADS.

To quantify how strongly participants' mental health problems relate to stressor exposure, we then calculate the relationship between E and P in the analysis sample for a given study phase by fitting a linear mixed model to predict P by E over all included participants and monitoring time points Tx, with random slopes and intercepts for participants. The E-P regression line is then determined by the fixed effect estimates for the E-P slope and intercept and serves as the normative E-P relationship for the analysis sample over the study phase of interest. The form of regression that explains most variance in P (linear or quadratic) will be used. At each Tx, we enter participants' individual E scores into the normative E-P line equation, giving us their expected P score when assuming normal stressor reactivity. The SR score is the individual's average residual onto the regression line for the study phase of interest.

On this basis, the outcome short-term stress resilience is defined as the average inverse SR score in the 12 weeks after the EM phase (early follow-up, corresponding to online monitoring time points T4 to T6); the outcome long-term stress resilience is defined as the average inverse SR score in the 24 weeks after the EM phase (whole follow-up, corresponding to time points T4 to T9).

### Quantification of BBB integrity

A pseudo-continuous ASL (pcASL) MRI sequence will be used to estimate BBB permeability. By reading out pcASL signals at different post-labeling delays (PLD) with multi-echo times, T2 values of intravascular and extravascular water can be estimated and subsequently fitted into a model to estimate the water exchange rate (Texch) from blood to brain tissue, an index quantifying BBB permeability. Analysis will be performed with the ExploreASL toolbox (https://sites.google.com/view/exploreasl), which includes image reconstruction, preprocessing (motion-correction, co-registration, skull-stripping), and Texch model fitting. Whole-brain BBB permeability will be quantified by averaging kw values across a whole-brain mask created from each participant’s T1 anatomical MRI image. As an auxiliary BBB permeability measure, data generated by the DP-pcASL sequence will be processed by a custom toolbox [20] to estimate water exchange rate (kw), a metric similar to Texch, to validate our main results.

On this basis, the outcome whole-brain BBB integrity at time points T0 (baseline) and T3 (end of EM phase) is defined as inverse whole-brain BBB permeability at these time points.

### Endpoints

The primary endpoint, relating to sub-objective O1a, is the prospective association between whole-brain BBB integrity at T0 and T3 and short-term resilience, taking into account the experimental manipulation (EM).

Key secondary endpoints relate to sub-objectives O1b and O1c and consist in, one, the effect of EM on whole-brain BBB integrity at T3, taking into account BBB integrity at T0; and, two, the mediation by the effect of EM on whole-brain BBB integrity at T3 of a potential effect of EM on short-term stress resilience.

Further secondary endpoints relate to sub-objectives O2a and O2b and consist in, one, the prospective association between whole-brain BBB integrity at T0 and T3 and long-term stress resilience, taking into account the experimental manipulation; and, two, the mediation by the effect of EM on whole-brain BBB integrity at T3 of a potential effect of EM on long-term stress resilience.

### Statistical methods

#### Data management

Data collection and data management will be conducted in compliance with the principles of the declaration of Helsinki (1996) and relevant national and regional regulations and will only follow study protocols approved by the regional ethics committee at each partner site/country. The principal investigator (PI) at each partner site will be responsible for data management in accordance with these principles. The PIs are further responsible for keeping records of experiments and data collection in line with good laboratory practice.

We will collect and process MRI imaging data (digital: DICOM), biosamples (blood), biological data from the analysis of biosamples (digital: concentrations of plasma proteins/peptides, RNAs, metabolites, and other molecules), and computerized (digital: offline and online collection) and paper-and-pencil (analogue) questionnaire and interview data.

In general, data storage will be locally for raw data and on central data storage facilities for processed and quality-assured data. Online monitoring data will be collected on the secured SoSci Survey platform and exported directly to a secured server located at, and administered by, LIR. Biosamples will be shipped by courier to UM (Biobank) for central analysis. Full data security is assured by the participating institutions.

#### Sample size calculation

Because the association of BBB function in stressor-exposed individuals at risk for stress-related mental health problems with stress resilience has never been investigated, we base our power calculation for the primary endpoint on the sensitivity to detect a medium effect size of Cohen’s d = 0.3. With a power of 0.8, a two-sided alpha-level of 0.05, and a linear mixed model with eight predictor variables (3 variables of interest and 5 covariates), we need n=109 participants. Taking into account 10% attrition, we aim to recruit a total of 122 participants (61 in each group).

#### Analysis populations

The primary analysis population will be the intention-to-treat (ITT) population (all participants who signed informed consent and were randomized). Secondary analyses will be conducted in the per-protocol population (ITT population without major protocol violations, not further described). No interim analysis will be performed.

#### Protocol violation

Key criteria to assess protocol violations are adherence to the EM, adherence to the online monitoring, and the completeness and quality of neuroimaging of BBB integrity. Adherence to the EM will be assessed based on a participant diary (paper and pencil) and the rendition of study drug blisters after the end of the EM at study visit 2 (T3). Adherence to online monitoring at a given online monitoring time point (T0-T9) will be assessed based on a criterion of full completion of the PHQ-ADS and MIMIS lists with reasonable scores (e.g., not all item scores in a list identical or zero) at that time point. Responses at online monitoring time points not fulfilling this criterion will be considered incomplete (missing). ME-pcASL images will be screened for excessive head movement as quality control measures. Additionally, fitted model parameters (e.g., CBF, ATT) resulting from ME-pcASL images will be screened for significant deviations from the reported values in the literature to ensure the reliability of model fitting.

#### Significance testing

All hypotheses will be tested on a two-sided level of significance α=0.05. The primary hypothesis (primary analysis relating to the primary sub-objective O1a) will be tested separately and without correction for multiple testing. Correction for multiple testing will be applied family-wise to the key secondary hypotheses (two key secondary analyses relating to the primary sub-objectives O1b and O1c), provided that the conditions to test the key secondary hypothesis relating to sub-objective O1c are met.

### Statistical analysis plan

#### General methods

All analyses will be conducted by means of linear mixed models. Depending on the pattern and extent of missing data, we will consider imputation approaches to ensure sufficient analysis power, especially in the analysis of SR at follow-up periods. We will perform autoregression-informed imputation, given that sufficient autocorrelation is present and if there is no evidence for non-randomly missing data.

Age, gender, site, and BMI will be used as covariates in all analyses. Selection of further covariates will follow a procedure used in earlier studies [47–49], such that all potential covariates with a p value <0.2 in univariate regression models on the endpoint will be included. We expect this to result in the selection of one additional covariate, most likely childhood trauma. Hence, in total, we expect to use five covariates.

#### Primary analysis (relating to primary sub-objective O1a)

To test for a prospective association of whole-brain BBB integrity with short-term stress resilience (inverse SR(T4-T6)), a linear mixed model will be used to regress whole-brain BBB integrity at baseline (BBB(T0)), whole-brain BBB integrity at the end of EM (BBB(T3)), EM (groups metformin and placebo), and covariates onto SR(T4-T6).

#### Key secondary analyses (relating to primary sub-objectives O1b and O1c)

To test for an effect of EM on whole-brain BBB integrity at end of EM (BBB(T3)), a linear mixed model will be used to regress EM, BBB(T0), and covariates onto BBB(T3).

If BBB(T3) shows a significant association with SR(T4-T6) (see O1a) and if EM shows a significant association with BBB(T3) (see O1b), we will test for mediation by the effect of EM on whole-brain BBB integrity at the end of EM (BBB(T3)) of a potential effect of EM on short-term stress resilience. Mediation analysis will be conducted using a Baron-Kenny approach with EM as predictor, BBB(T3) as mediator, SR(T4-T6) as outcome, and BBB(T0) and earlier covariates as covariates.

#### Further secondary analyses (relating to secondary sub-objectives O2a and O2b)

To test for a prospective association of whole-brain BBB permeability with long-term stress resilience (inverse SR(T4-T9)), a linear mixed model will be used to regress BBB(T0), BBB(T3), EM, and covariates onto SR(T4-T9).

If BBB(T3) shows a significant association with SR(T4-T9) (see O2a) and if EM shows a significant association with BBB(T3) (see O1b), we will test for mediation by the effect of EM on whole-brain BBB at the end of EM (BBB(T3)) of a potential effect of EM on long-term stress-resilience. Mediation analysis will be conducted using a Baron-Kenny approach with EM as predictor, BBB(T3) as mediator, SR(T4-T9) as outcome, and BBB(T0) and earlier covariates as covariates.

#### Additional analyses

Additional exploratory analyses will assess the relationships between socio-demographic and anamnestic variables, psycho-social variables, blood-based molecular and cellular markers of immune, metabolic, and BBB function, and additional neuroimaging variables and the study outcomes. As additional study outcomes, regional measures of BBB integrity (esp. of hippocampus, ventral striatum, and prefrontal cortex) as well as blood-based molecular markers of BBB function will also be used. Measures of BBB function obtained from the ME-pcASL sequence will be compared with measures obtained from the DP-pcASL sequence and potential deviations will be discussed and assessed for statistical effects.

## Discussion

The study has the potential to establish for the first time a relationship between BBB function and stress resilience in humans and to yield hints at a potential causal role of BBB function in resilience. If a role of the BBB in stress resilience can be shown, methods to enhance BBB function under stress could in principle be exploited for resilience promotion, that is, for the prevention of stress-related mental health problems in at-risk groups. Such methods may include pharmacological augmentation, for instance with metformin. Another future way to exploit the anticipated results is to use an assessment of BBB function, via MRI or blood-based markers, as a precision medicine tool allowing for identifying individuals with heightened risk and/or for targeting BBB-protective interventions specifically at individuals with impaired BBB function. Future follow-up work exploiting the anticipated results will require clinical efficacy trials.

## Supplementary Information

Supplementary Material 1: Appendix 1 contains a list of the questionnaires used to collect socio-demographic and anamnestic information at online pre-screening and online as part of the screening visit, and the corresponding sources.

Supplementary Material 2: Appendix 2 contains a list of the online questionnaires used to collect information on stressor exposure, mental health, and psycho-social variables at the online monitorings and the study visits, and the corresponding sources.

Supplementary Material 3: Appendix 3 contains detailed descriptions of the MRI scanning sequences.

Supplementary Material 4: Appendix 4 contains the Case Report Form (CRF)

Supplementary Material 5: Appendix 5 contains the SPIRIT 2025 checklist [50]

## Data Availability

No datasets were generated or analysed during the current study.

## Abbreviations

AE: Adverse Event
ASL: Arterial Spin Labelling
BBB: Blood Brain Barrier
BDI: Beck’s Depression Inventory
BID: Bis In Die (twice a day)
BL: Baseline
BMFTR: Bundesministerium für Forschung, Technologie und Raumfahrt
BMI: Body Mass Index
CBF: Core Binding Factor
CHF: Confoederatio HelveticaFranc (Swiss Franc)
CRF: Case Report Form
CRP: C-Reactive Protein
CSD: Chronic Social Defeat
CSF: Cerebrospinal Fluid
C-SSRS: Columbia Suicide Severity Rating Scale
DH: Daily Hassle
DSMB: Data Safety MonitoringBoard
DUAC: Data Use and Access Committee
E: Stressor Exposure
EM: Experimental Manipulation
EoEM: End of Experimental Manipulation
EoFU: End of Follow Up
EU: European Union
FU: Follow Up
GCP: Good Clinical Practice
GHQ: General Health Questionnaire
HPC: Hippocampus
ICAM: Intercellular Adhesion Molecule
IL: Interleukin
ITT: Intention to Treat
LE: Life Events
LEQ: Life Event Questionnaire
LIR: Leibniz Institute for Resilience Research
M.I.N.I.: Mini-International Neuropsychiatric Interview
MAPK: Mitogen-activated protein kinase
MDD: Major Depressive Disorder
MIMIS: Mainz Inventory of Microstressors
MMSE: Mini-Mental State Examination
MoFU: Mid of Follow Up
MRI: Magnetic Resonance Imaging
mTOR: Mammalian Target Of Rapamycin
NIC: Neuroimaging Centre
OSF: Open Science Framework
P: Mental Health Problem
PFC: Prefrontral Cortex
PHQ-ADS: Patient Health Questionnaire Anxiety and Depression Scale
PI: Principal Investigator
PLD: Post-labeling delays
PLN: Polish New złoty
SAA: Serum amyloid A
SAE: Serious Adverse Event
SR: Stressor Reactivity
SUSAR: Suspected Unexpected Serious Adverse Reaction
TNF: Tumor Necrosis Factor
UM: Universitätsmedizin Mainz
VCAM-1: Vascular Cell Adhesion Molecule 1
VS: Ventral Striatum
VWF: Von Willebrand factor

## References

[1] Global, regional, and, national, burden, of, 12, mental, disorders, in, 204, countries, and, territories, 1990–2019:, a, systematic, analysis, for, the, Global, Burden, of, Disease, Study, 2019. Lancet Psychiatry. 2022;9(2):137–50. 10.1016/S2215-0366(21)00395-3 PMC8776563 35026139

[2] COVID-19 Mental Disorders Collaborators. Global prevalence and burden of depressive and anxiety disorders in 204 countries and territories in 2020 due to the COVID-19 pandemic. Lancet. 2021;398(10312):1700–12. 34634250 10.1016/S0140-6736(21)02143-7 PMC8500697

[3] Calcia MA, Bonsall DR, Bloomfield PS, Selvaraj S, Barichello T, Howes OD. Stress and neuroinflammation: a systematic review of the effects of stress on microglia and the implications for mental illness. Psychopharmacology. 2016;233(9):1637–50. 26847047 10.1007/s00213-016-4218-9 PMC4828495

[4] Jorm AF, Patten SB, Brugha TS, Mojtabai R. Has increased provision of treatment reduced the prevalence of common mental disorders? Review of the evidence from four countries. World Psychiatry. 2017;16(1):90–9. 28127925 10.1002/wps.20388 PMC5269479

[5] Kalisch R, Baker DG, Basten U, Boks MP, Bonanno GA, Brummelman E et al. The resilience framework as a strategy to combat stress-related disorders. Nat Hum Behav [Internet]. 2017;1(11):784. Available from: https://www.nature.com/articles/s41562-017-0200-8 10.1038/s41562-017-0200-8 31024125

[6] Stelmach R, Kocher EL, Kataria I, Jackson-Morris AM, Saxena S, Nugent R. The global return on investment from preventing and treating adolescent mental disorders and suicide: a modelling study. BMJ Glob Health. 2022. 10.1136/bmjgh-2021-007759. 35705224 10.1136/bmjgh-2021-007759 PMC9240828

[7] Penninx BWJH, Benros ME, Klein RS, Vinkers CH. How COVID-19 shaped mental health: from infection to pandemic effects. Nat Med [Internet]. 2022;28(10):2027–37. Available from: https://www.ncbi.nlm.nih.gov/pmc/articles/PMC9711928/10.1038/s41591-022-02028-2 PMC9711928 36192553

[8] Tupler LA, Hong JY, Gibori R, Blitchington TF, Krishnan KRR. Suicidal ideation and sex differences in relation to 18 major psychiatric disorders in college and university students: anonymous web-based assessment. J Nerv Ment Dis. 2015;203(4):269–78. 25784307 10.1097/NMD.0000000000000277

[9] Galea I, Bechmann I, Perry VH. What is immune privilege (not)? Trends Immunol. 2007;28(1):12–8. 17129764 10.1016/j.it.2006.11.004

[10] Louveau A, Harris TH, Kipnis J. Revisiting the mechanisms of CNS immune privilege. Trends Immunol. 2015;36(10):569–77. 26431936 10.1016/j.it.2015.08.006 PMC4593064

[11] Abbott NJ, Patabendige AAK, Dolman DEM, Yusof SR, Begley DJ. Structure and function of the blood-brain barrier. Neurobiol Dis. 2010;37:13–25. 19664713 10.1016/j.nbd.2009.07.030

[12] Danielski LG, Giustina A, Della, Badawy M, Barichello T, Quevedo J, Dal-Pizzol F, et al. Brain barrier breakdown as a cause and consequence of neuroinflammation in sepsis. Mol Neurobiol. 2018;55(2):1045–53. 28092082 10.1007/s12035-016-0356-7

[13] Cathomas F, Holt LM, Parise EM, Liu J, Murrough JW, Casaccia P, et al. Beyond the neuron: role of non-neuronal cells in stress disorders. Neuron. 2022;110(7):1116–38. 35182484 10.1016/j.neuron.2022.01.033 PMC8989648

[14] Powell ND, Sloan EK, Bailey MT, Arevalo JMG, Miller GE, Chen E, et al. Social stress up-regulates inflammatory gene expression in the leukocyte transcriptome via β-adrenergic induction of myelopoiesis. Proc Natl Acad Sci U S A. 2013;110(41):16574–9. 24062448 10.1073/pnas.1310655110 PMC3799381

[15] Menard C, Pfau ML, Hodes GE, Kana V, Wang VX, Bouchard S, et al. Social stress induces neurovascular pathology promoting depression. Nat Neurosci. 2017;20(12):1752–60. 29184215 10.1038/s41593-017-0010-3 PMC5726568

[16] Dudek KA, Dion-Albert L, Lebel M, LeClair K, Labrecque S, Tuck E, et al. Molecular adaptations of the blood-brain barrier promote stress resilience vs. depression. Proc Natl Acad Sci U S A. 2020;117(6):3326–36. 10.1073/pnas.1914655117. 31974313 10.1073/pnas.1914655117 PMC7022213

[17] Rebeles F, Fink J, Anzai Y, Maravilla KR. Blood-brain barrier imaging and therapeutic potentials. Top Magn Reson Imaging. 2006;17(2):107–16. 17198226 10.1097/RMR.0b013e31802f5df9

[18] Marchi N, Rasmussen P, Kapural M, Fazio V, Kight K, Mayberg MR, et al. Peripheral markers of brain damage and blood-brain barrier dysfunction. Restor Neurol Neurosci. 2003;21(3–4):109–21. 14530574 PMC4066375

[19] Heye AK, Culling RD, Valdés Hernández MDC, Thrippleton MJ, Wardlaw JM. Assessment of blood-brain barrier disruption using dynamic contrast-enhanced MRI. A systematic review. Neuroimage Clin. 2014;6:262–74. 25379439 10.1016/j.nicl.2014.09.002 PMC4215461

[20] Shao X, Ma SJ, Casey M, D’Orazio L, Ringman JM, Wang DJJ. Mapping water exchange across the blood-brain barrier using 3D diffusion-prepared arterial spin labeled perfusion MRI. Magn Reson Med. 2019;81(5):3065–79. 30561821 10.1002/mrm.27632 PMC6414249

[21] Joseph CR. Utilizing 3D arterial spin labeling to identify cerebrovascular leak and glymphatic obstruction in neurodegenerative disease. Diagnostics (Basel). 2021;11(10):1888. 34679586 10.3390/diagnostics11101888 PMC8534509

[22] Andersson M, Alvarez-Cermeño J, Bernardi G, Cogato I, Fredman P, Frederiksen J, et al. Cerebrospinal fluid in the diagnosis of multiple sclerosis: a consensus report.Journal of Neurology, Neurosurgery & Psychiatry [Internet]. 1994;57(8):897. Available from: http://jnnp.bmj.com/content/57/8/897.abstract. http://jnnp.bmj.com/content/57/8/897.abstract 10.1136/jnnp.57.8.897 PMC1073070 8057110

[23] Gudmundsson P, Skoog I, Waern M, Blennow K, Pálsson S, Rosengren L, et al. The relationship between cerebrospinal fluid biomarkers and depression in elderly women. Am J Geriatr Psychiatry. 2007;15(10):832–8. 17911361 10.1097/JGP.0b013e3180547091

[24] Niklasson F, Agren H. Brain energy metabolism and blood-brain barrier permeability in depressive patients: analyses of creatine, creatinine, urate, and albumin in CSF and blood. Biol Psychiatry. 1984;29:1183–206. 6498242

[25] Kanner AA, Marchi N, Fazio V, Mayberg MR, Koltz MT, Siomin V, et al. <article-title update="added">Serum S100β: *A noninvasive marker of blood‐brain barrier function and brain lesions*. Cancer. 2003;97(11):2806–13. 12767094 10.1002/cncr.11409 PMC4135471

[26] PolyakovaM, Sander C, Arelin K, Lampe L, Luck T, Luppa M et al. First evidence for glial pathology in late life minor depression: S100b is increased in males with minor depression. Front Cell Neurosci. 2015;9(OCT). 10.3389/fncel.2015.00406. 10.3389/fncel.2015.00406 PMC4598479 26500502

[27] Dion-Albert L, Cadoret A, Doney E, Kaufmann FN, Dudek KA, Daigle B, et al. Vascular and blood-brain barrier-related changes underlie stress responses and resilience in female mice and depression in human tissue. Nat Commun. 2022;13(1):164. 35013188 10.1038/s41467-021-27604-x PMC8748803

[28] Janssen EPCJ, Köhler S, Geraets AFJ, Stehouwer CDA, Schaper NC, Sep SJS, et al. Low-grade inflammation and endothelial dysfunction predict four-year risk and course of depressive symptoms: the Maastricht study. Brain Behav Immun. 2021;97:61–7. 34186200 10.1016/j.bbi.2021.06.013

[29] Vennin C, Hewel C, Todorov H, Wendelmuth M, Radyushkin K, Heimbach A, et al. A resilience related glial-neurovascular network is transcriptionally activated after chronic social defeat in male mice. Cells. 2022;11(21):3405. 36359800 10.3390/cells11213405 PMC9655779

[30] Wullschleger S, Loewith R, Hall MN. TOR signaling in growth and metabolism. Cell. 2006;124:471–84. 16469695 10.1016/j.cell.2006.01.016

[31] Liu GY, Sabatini DM. mTOR at the nexus of nutrition, growth, ageing and disease. Nat Reviews Mol Cell Biology Nat Res. 2020;21:183–203. 10.1038/s41580-019-0199-y PMC7102936 31937935

[32] Lin AL, Zheng W, Halloran JJ, Burbank RR, Hussong SA, Hart MJ, et al. Chronic rapamycin restores brain vascular integrity and function through NO synthase activation and improves memory in symptomatic mice modeling Alzheimer’s disease. J Cereb Blood Flow Metab. 2013;33(9):1412–21. 23801246 10.1038/jcbfm.2013.82 PMC3764385

[33] Lin AL, Jahrling JB, Zhang W, Derosa N, Bakshi V, Romero P, et al. Rapamycin rescues vascular, metabolic and learning deficits in Apolipoprotein E4 Transgenic mice with pre-symptomatic alzheimer’s disease. J Cereb Blood Flow Metab. 2017;37(1):217–26. 26721390 10.1177/0271678X15621575 PMC5167110

[34] Van Skike CE, Jahrling JB, Olson AB, Sayre NL, Hussong SA, Ungvari Z, et al. Inhibition of mTOR protects the blood-brain barrier in models of alzheimer’s disease and vascular cognitive impairment. Am J Physiol Heart Circ Physiol [Internet]. 2018;314:693–703. Available from: www.ajpheart.org. 10.1152/ajpheart.00570.2017 PMC5966773 29351469

[35] Chi OZ, Kiss GK, Mellender SJ, Liu X, Weiss HR. Rapamycin decreased blood-brain barrier permeability in control but not in diabetic rats in early cerebral ischemia. Neurosci Lett. 2017;654:17–22. 28625574 10.1016/j.neulet.2017.06.021

[36] HadleyG, Beard DJ, Couch Y, Neuhaus AA, Adriaanse BA, DeLuca GC, et al. Rapamycin in ischemic stroke: old drug, new tricks? Journal of Cerebral Blood Flow and Metabolism. Volume 39. SAGE Publications Ltd; 2019. pp. 20–35. 10.1177/0271678X18807309. 10.1177/0271678X18807309 PMC6311672 30334673

[37] Chen S, Gan D, Lin S, Zhong Y, Chen M, Zou X, et al. Metformin in aging and aging-related diseases: clinical applications and relevant mechanisms. Theranostics. 2022;12(6):2722–40. 35401820 10.7150/thno.71360 PMC8965502

[38] Triggle CR, Mohammed I, Bshesh K, Marei I, Ye K, Ding H, et al. Metformin: is it a drug for all reasons and diseases? Metabolism. 2022;133:155223. 35640743 10.1016/j.metabol.2022.155223

[39] Amin S, Lux A, O’Callaghan F. The journey of metformin from glycaemic control to mTOR inhibition and the suppression of tumour growth. Br J Clin Pharmacol. 2019;85(1):37–46. 30290005 10.1111/bcp.13780 PMC6303203

[40] Bai B, Chen H. Metformin: a novel weapon against inflammation. Front Pharmacol. 2021;12:622262. 33584319 10.3389/fphar.2021.622262 PMC7880161

[41] Liu Y, Tang G, Li Y, Wang Y, Chen X, Gu X, et al. Metformin attenuates blood-brain barrier disruption in mice following middle cerebral artery occlusion. J Neuroinflammation. 2014;11:177. 25315906 10.1186/s12974-014-0177-4 PMC4201919

[42] Leiner DJ. SoSci Survey (Version 3.6.11) [Internet]. 2024 [cited 2025 Apr 18]. Available from: https://www.soscisurvey.de

[43] Cuthbert BN, Insel TR. Toward the future of psychiatric diagnosis: the seven pillars of RDoC. BMC Med. 2013;11:126. 23672542 10.1186/1741-7015-11-126 PMC3653747

[44] Mahroo A, Buck MA, Huber J, Breutigam NJ, Mutsaerts HJMM, Craig M, et al. Robust multi-TE ASL-based blood–brain barrier integrity measurements. Front Neurosci. 2021. 10.3389/fnins.2021.719676. 34924924 10.3389/fnins.2021.719676 PMC8678075

[45] Shao X, Jann K, Ma SJ, Yan L, Montagne A, Ringman JM, et al. Comparison between blood-brain barrier water exchange rate and permeability to Gadolinium-Based contrast agent in an elderly cohort. Front Neurosci. 2020. 10.3389/fnins.2020.571480. 33328848 10.3389/fnins.2020.571480 PMC7733970

[46] Kalisch R, Köber G, Binder H, Ahrens KF, Basten U, Chmitorz A, et al. The frequent stressor and mental health monitoring-paradigm: a proposal for the operationalization and measurement of resilience and the identification of resilience processes in longitudinal observational studies. Front Psychol. 2021. 10.3389/fpsyg.2021.710493. 34539510 10.3389/fpsyg.2021.710493 PMC8444985

[47] BögemannSA, Puhlmann LMC, Wackerhagen C, Zerban M, Riepenhausen A, Köber G et al. Psychological resilience factors and their association with weekly stressor reactivity during the COVID-19 outbreak in europe: prospective longitudinal study. JMIR Ment Health. 2023;10(1). 10.2196/46518. 10.2196/46518 PMC10618882 37847551

[48] Zerban M, Puhlmann LMC, Lassri D, Fonagy P, Montague PR, Kiselnikova N, et al. What helps the helpers? Resilience and risk factors for general and profession-specific mental health problems in psychotherapists during the COVID-19 pandemic. Front Psychol. 2023;14:1272199. 38164261 10.3389/fpsyg.2023.1272199 PMC10757941

[49] Veer IM, Riepenhausen A, Zerban M, Wackerhagen C, Puhlmann LMC, Engen H, et al. Psycho-social factors associated with mental resilience in the Corona lockdown. Transl Psychiatry. 2021;11(1):67. 33479211 10.1038/s41398-020-01150-4 PMC7817958

[50] ChanAW, Boutron I, Hopewell S, Moher D, Schulz KF, Collins GS et al. SPIRIT 2025 statement: updated guideline for protocols of randomised trials. BMJ. 2025;389. 10.1371/journal.pmed.1004589. 10.1136/bmj-2024-081477 PMC12035670 40294953

