# Supplementary material for: Study protocol: role of the blood-brain barrier in stress resilience: investigating new pathways towards Pharmacological augmentation of stress resilience (a PHASR-PP project study)
Source: BMC Psychol. 2026 Mar 17;14:486. doi: 10.1186/s40359-026-04118-z (PMC13063912; doi:10.1186/s40359-026-04118-z)
Supplement: Supplementary file 2 — Supplementary Material 2: Appendix 2 contains a list of the online questionnaires used to collect information on stressor exposure, mental health, and psycho-social variables at the online monitorings and the study visits, and the corresponding sources. [file 40359_2026_4118_MOESM2_ESM.docx]

PHASR-PP

Role of the blood-brain barrier in stress resilience: investigating new pathways towards pharmacological augmentation of stress resilience

Case Report File (CRF)

Participant ID:

_________________________

# 1 Pre-Screening

## Inclusion criteria

The pre-screening is conducted online via Sosci Survey.

Did the participant meet all of the inclusion criteria? ☐ yes ☐ no

If no -> exclusion

Did the participant meet any of the exclusion criteria? ☐ yes ☐ no

If yes -> exclusion

Is it safe for the participant to take Metformin? ☐ yes ☐ no

If no -> exclusion

Did the participant pass the MRI screening? ☐ yes ☐ no

If no -> exclusion

What was the GHQ-28 score of the participant? ____________________

In conclusion; can the participant be included? ☐ yes ☐ no

If the participant is excluded, please elaborate why:

___________________________________________________________________________________________

___________________________________________________________________________________________

___________________________________________________________________________________________

If all inclusion criteria are met and no exclusion criteria apply, the subject has the opportunity to contact us. If they are interested in participating, send the study information via email and ask them to respond if they wish to take part. Then, schedule an appointment for the screening visit and note the planned date and time.

Study information sent via mail ☐ yes ☐ no

Date and time the mail was sent:_______________________________________________________________

At least 24 hours must have passed before the subject can declare interest in participating in the study.

Subject is interested in participating in the study ☐ yes ☐ no

Date and time the subject has declared interest to participate:________________________________________

Scheduled date and time for the screening visit:____________________________________________________

# 2 Screening Visit

Provide participant Information and Consent Forms. Ask the participant to sign the Consent Forms after all questions are clarified. Ask the participant to re-create their pre-screening ID.

The subject has signed the participant information and provided written informed consent.

☐ yes ☐ no

Vital signs (BP, pulse, temperature) ☐ yes ☐ no

BP: ……./……. HR: …….

Clinical signs of infection ☐ yes ☐ no

## Blood Sample

| **Sampling Date (DD/MM/YY)** |  |
| --- | --- |
| **Sampling Time** |  |
| **Medical technician** |  |
| **Measures Taken*** |  |
| **Comments** |  |

***Amount of blood taken (tubes) and for which purpose.**

Creatinin Clearence results:____________________________________________________________________

HBA1c results:____________________________________________________________________

## Drug test (Urine)

| **Sampling Date (DD/MM/YY)** |  |
| --- | --- |
| **Sampling Time** |  |
| **Test Result** | Positive ☐ Negative ☐ |

The participant is free of psychoactive drugs as assessed by urine tests. ☐ yes ☐ no

## Pregnancy test

| **Sampling Date (DD/MM/YY)** |  |
| --- | --- |
| **Sampling Time** |  |
| **Test Result** | Positive ☐ Negative ☐ |

The participant is not pregnant as assessed by urine tests. ☐ yes ☐ no

## Psychological Assessment

MMSE - Mini-Mental-Status-Examination passed? ☐ yes ☐ no

___________________________

MMSE Score

Beck Depression Inventory (BDI) passed?* ☐ yes ☐ no

___________________________

BDI Score

Columbia-Suicide Severity Rating Scale (C-SSRS) passed?* ☐ yes ☐ no

___________________________

C-SSRS Score

Three or more adverse life events (LEQ)? ☐ yes ☐ no

___________________________

Life events

Demographics information ☐ yes ☐ no

Answers: ______________________________________________________

______________________________________________________

Medical History examined ☐ yes ☐ no

**Comments**: ___________________________________________________________________________________________

___________________________________________________________________________________________

Concomitant Medication and Medical Conditions examined ☐ yes ☐ no

**Comments**: ___________________________________________________________________________________________

___________________________________________________________________________________________

Mini International Neuropsychiatric Interview (M.I.N.I) diagnosis?* ☐ yes ☐ no

M.I.N.I diagnostic criteria _____________________________________________________

(excluding the participant)

*If the subjects shows signs of suicidal or self-harming thoughts, they will be advised to seek support or help, including the phone number of the local suicide-helpline.

## Screening Conclusion (preliminary)

Termination ☐ yes ☐ no

Reason for termination:_______________________________________________________________________

Date and Time:______________________________________________________________________________

## Visit Schedule

Schedule the visits for the rest of the study. Make sure visit 1 and visit 2 are 12 weeks apart, and visit 2 and visit 3 are 24 weeks apart.

Scheduled date and time for Visit 1:_____________________________________________________________

Scheduled date and time for Visit 2:_____________________________________________________________

Scheduled date and time for Visit 3:_____________________________________________________________

## Remuneration

The participant has received the remuneration for the Screening visit in the amount of 60 Swiss Francs / 40 Euro / 30 PLN.

☐ yes ☐ no

The participant has signed the remuneration receipt.

☐ yes ☐ no

# 3 Study Visit 1 (T0)

## Magnetic resonance imaging (MRI)

Participant has signed the written informed consent for the MRI ☐ yes ☐ no

Participant was instructed regarding MRI procedure and safety ☐ yes ☐ no

| Date | Time (MRI In) | Time (MRI Out) |
| --- | --- | --- |
|  |  |  |

**All sequences measured:**

T1-weighted MPRAGE ☐ yes ☐ no

Multi-TE pseudo-continuous (ME-pcASL) ☐ yes ☐ no

Multi-echo multi-band EPI for rsfMRI ☐ yes ☐ no

T2-weighted FLAIR ☐ yes ☐ no

Diffusion prepared pseudo-continuous (DP-pcASL) ☐ yes ☐ no

**Comments MRI:**

____________________________________________________________________________________________________________________________________________________________________________________________________________________________________________________________________________________________________________________________________________________________________________

## Questionnaire battery

| **No.** | **Questionnaire** | **Fully Answered** | |
| --- | --- | --- | --- |
| 1 | Anxiety Sensitivity Index (ASI) | ☐ yes | ☐ no |
| 3 | Brief Resilience Scale (BRS) | ☐ yes | ☐ no |
| 4 | Cognitive Emotion Regulation Questionnaire (CERQ-short) | ☐ yes | ☐ no |
| 5 | Cognitive Orientation to Problems Experienced questionnaire (Brief COPE) | ☐ yes | ☐ no |
| 6 | Coping Flexibility Scale Revised Questionnaire (CFS-R) | ☐ yes | ☐ no |
| 7 | Context Sensitivity Index (CSI) | ☐ yes | ☐ no |
| 7 | Difficulties in Emotion Regulation Questionnaire (DERS) | ☐ yes | ☐ no |
| 8 | Flexible Emotion Regulation Scale (FlexER-10 – Scale) | ☐ yes | ☐ no |
| 10 | General Self Efficacy Scale (GSE) | ☐ yes | ☐ no |
| 11 | Internal External Locus of Control – 4 (IE-4) | ☐ yes | ☐ no |
| 12 | Life Events Questionnaire (LEQ) | ☐ yes | ☐ no |
| 13 | Life Orientation Test – Revised (LOT-R) | ☐ yes | ☐ no |
| 15 | Maltreatment and Abuse Chronology of Exposure (MACE) | ☐ yes | ☐ no |
| 16 | NEO-FFI – Agreeableness | ☐ yes | ☐ no |
| 17 | NEO-FFI – Neuroticism | ☐ yes | ☐ no |
| 18 | Oslo 3 Item Social Support Scale (OSSS-3) | ☐ yes | ☐ no |
| 19 | Perceived Positive Appraisal Style Scale; content-focused (PASS-content) | ☐ yes | ☐ no |
| 20 | Perceived Positive Appraisal Style Scale; process-focused (PASS-process) | ☐ yes | ☐ no |
| 21 | Perceived Social Status Scale (PSS-10) | ☐ yes | ☐ no |
| 23 | Revised Symptom Checklist 90 (SCL-90-R)* | ☐ yes | ☐ no |
| 24 | State Trait Anxiety Inventory - trait (STAI-T) | ☐ yes | ☐ no |
| 25 | Psychological Flexibility Questionnaire (PFQ) | ☐ yes | ☐ no |
| 26 | Visual Imagery Vividness (VVIQ) | ☐ yes | ☐ no |
| 28 | Pittsburgh Sleep Quality Index (PSQI) | ☐ yes | ☐ no |

*If the subjects shows signs of suicidal or self-harming thoughts, they will be re-directed to a page advising them to seek support or help, including the phone number of the local suicide-helpline.

**Comments Questionnaires:**

__________________________________________________________________________________________________________________________________________________________________________________________________________________________________________________________________________________________________________________________________________________________________________________________________________________________________________________________________________________________________________________________________________________________________

## Study Drug distribution and monitoring

The participant has received an explanation regarding the intake of the study drug.

☐ yes ☐ no

The participant has been handed over the study drug.

☐ yes ☐ no

The participant has received an explanation regarding the online monitoring and all open questions were answered.

☐ yes ☐ no

Termination ☐ yes ☐ no

Reason for termination:_______________________________________________________________________

Date and Time:______________________________________________________________________________

## Remuneration

The participant has received the remuneration for Visit 1 in the amount of 80 Swiss Francs / 50 Euro / 240 PLN.

☐ yes ☐ no

The participant has signed the remuneration receipt.

☐ yes ☐ no

## Adverse Events

Were there any adverse events during this visit? ☐ yes ☐ no

If so, please elaborate:________________________________________________________________________

___________________________________________________________________________________________

___________________________________________________________________________________________

## Continuation of the Study

Is there any reason to discontinue the participation in the study? ☐ yes ☐ no

If so, why: _________________________________________________________________________________

Otherwise, confirmation next visits:

Scheduled date and time for visit 2:______________________________________________________________

Scheduled date and time for visit 3:______________________________________________________________

# 4 Online Monitoring (T1-T3)

## Monitoring Questionnaires

| **Timepoint** | **Questionnaire** | **Fully answered** | |
| --- | --- | --- | --- |
| T1 |  |  |  |
|  | Daily Hassle (MIMIS)* | ☐ yes | ☐ no |
|  | Life events (LEQ) | ☐ yes | ☐ no |
|  | PHQ-ADS** | ☐ yes | ☐ no |
|  | Sleep questionnaire*** | ☐ yes | ☐ no |
|  | Current Mood and Menstrual Cycle | ☐ yes | ☐ no |
|  |  |  |  |
| T2 | Daily Hassle (MIMIS) | ☐ yes | ☐ no |
|  | Life events (LEQ) | ☐ yes | ☐ no |
|  | PHQ-ADS | ☐ yes | ☐ no |
|  | Sleep questionnaire | ☐ yes | ☐ no |
|  | Current Mood and Menstrual Cycle | ☐ yes | ☐ no |
|  |  |  |  |
| T3 | Daily Hassle (MIMIS) | ☐ yes | ☐ no |
|  | Life events (LEQ) | ☐ yes | ☐ no |
|  | PHQ-ADS | ☐ yes | ☐ no |
|  | Sleep questionnaire | ☐ yes | ☐ no |
|  | Current Mood and Menstrual Cycle | ☐ yes | ☐ no |

* Mainz Inventory of Microstressors
** Patient Health Questionnaire - Anxiety and Depression Scale
*** Sleep Regularity Questionnaire + 2 items from the Pittsburgh Sleep Quality Index

**Comments Online Monitoring:**

__________________________________________________________________________________________

__________________________________________________________________________________________

__________________________________________________________________________________________

__________________________________________________________________________________________

___________________________________________________________________________________________

___________________________________________________________________________________________

## Medication Diary

For each medication moment, place a ‘✔’ if the dose was taken correctly or a ‘🗶’ if they missed or skipped it. Please clarify below when a dose was missed.

|  | Week 1 | Week 2 | Week 3 | Week 4 | Week 5 | Week 6 | Week 7 | Week 8 | Week 9 | Week 10 | Week 11 | Week 12 |
| --- | --- | --- | --- | --- | --- | --- | --- | --- | --- | --- | --- | --- |
| Day 1 |  |  |  |  |  |  |  |  |  |  |  |  |
| Day 2 |  |  |  |  |  |  |  |  |  |  |  |  |
| Day 3 |  |  |  |  |  |  |  |  |  |  |  |  |
| Day 4 |  |  |  |  |  |  |  |  |  |  |  |  |
| Day 5 |  |  |  |  |  |  |  |  |  |  |  |  |
| Day 6 |  |  |  |  |  |  |  |  |  |  |  |  |
| Day 7 |  |  |  |  |  |  |  |  |  |  |  |  |

**Comments Medication Diary**

___________________________________________________________________________________________________________________________________________________________________________________________________________________________________________________________________________________________________________________________________________________________________________________________________________________________________________________________________________________________________________________________________________________________________________________________________________________________________________________________________________________________________________________________________________________________________________________________________________________________________________________

## Adverse Events

Were there any adverse events during this period? ☐ yes ☐ no

If so, please elaborate:________________________________________________________________________

___________________________________________________________________________________________

___________________________________________________________________________________________

## Continuation of the Study

Is there any reason to discontinue the participation in the study? ☐ yes ☐ no

If so, why: _________________________________________________________________________________

## 5 Study Visit 2 (T3)

## Blood Sample

| **Sampling Date (DD/MM/YY)** |  |
| --- | --- |
| **Sampling Time** |  |
| **Medical technician** |  |
| **Measures Taken *** |  |
| **Comments** |  |

***Amount of blood taken (tubes) and for which purpose.**

Study drug blisters handed over ☐ yes ☐ no

Study drug leftovers ☐ yes ☐ no

**Comments on study drug leftovers:**

__________________________________________________________________________________________________________________________________________________________________________________________________________________________________________________________________________________________________________________________________________________________________________________________________________________________________________________________________________________________________________________________________________________________________

## Magnetic resonance imaging (MRI)

Participant has signed the written informed consent for the MRI ☐ yes ☐ no

Participant was instructed regarding MRI procedure and safety ☐ yes ☐ no

| Date | Time (MRI In) | Time (MRI Out) |
| --- | --- | --- |
|  |  |  |

**All sequences measured:**

T1-weighted MPRAGE ☐ yes ☐ no

Multi-TE pseudo-continuous (ME-pcASL) ☐ yes ☐ no

Multi-echo multi-band EPI for rsfMRI ☐ yes ☐ no

T2-weighted FLAIR ☐ yes ☐ no

Diffusion prepared pseudo-continuous (DP-pcASL) ☐ yes ☐ no

**Comments MRI:**

__________________________________________________________________________________________________________________________________________________________________________________________________________________________________________________________________________________________________________________________________________________________________________________________________________________________________________________________________________________________________________________________________________________________________

Termination ☐ yes ☐ no

Reason for termination:_______________________________________________________________________

Date and Time:______________________________________________________________________________

## Questionnaire Battery

| **No.** | **Questionnaire** | **Fully Answered** | |
| --- | --- | --- | --- |
| 1 | Anxiety Sensitivity Index (ASI) | ☐ yes | ☐ no |
| 3 | Brief Resilience Scale (BRS) | ☐ yes | ☐ no |
| 4 | Childhood Trauma Questionnaire - Short Form (CTQ-SF) | ☐ yes | ☐ no |
| 4 | Cognitive Emotion Regulation Questionnaire (CERQ-short) | ☐ yes | ☐ no |
| 5 | Cognitive Orientation to Problems Experienced questionnaire (Brief COPE) | ☐ yes | ☐ no |
| 6 | Coping Flexibility Scale Revised Questionnaire (CFS-R) | ☐ yes | ☐ no |
| 7 | Context Sensitivity Index (CSI) | ☐ yes | ☐ no |
| 7 | Difficulties in Emotion Regulation Questionnaire (DERS) | ☐ yes | ☐ no |
| 8 | Flexible Emotion Regulation Scale (FlexER-10 – Scale) | ☐ yes | ☐ no |
| 10 | General Self Efficacy Scale (GSE) | ☐ yes | ☐ no |
| 11 | Internal External Locus of Control – 4 (IE-4) | ☐ yes | ☐ no |
| 13 | Life Orientation Test – Revised (LOT-R) | ☐ yes | ☐ no |
| 17 | Oslo 3 Item Social Support Scale (OSSS-3) | ☐ yes | ☐ no |
| 18 | Perceived Positive Appraisal Style Scale; content-focused (PASS-content) | ☐ yes | ☐ no |
| 19 | Perceived Positive Appraisal Style Scale; process-focused (PASS-process) | ☐ yes | ☐ no |
| 20 | Perceived Social Status Scale (PSS-10) | ☐ yes | ☐ no |
| 22 | Revised Symptom Checklist 90 (SCL-90-R)* | ☐ yes | ☐ no |
| 23 | State Trait Anxiety Inventory - trait (STAI-T) | ☐ yes | ☐ no |
| 24 | Psychological Flexibility Questionnaire (PFQ) | ☐ yes | ☐ no |
| 27 | Pittsburgh Sleep Quality Index (PSQI) | ☐ yes | ☐ no |

*If the subjects shows signs of suicidal or self-harming thoughts, they will be re-directed to a page advising them to seek support or help, including the phone number of the local suicide-helpline.

## Remuneration

The participant has received the remuneration for Visit 2, the online monitoring and study drug take in the amount of 290 Swiss Francs / 290 euro / 880 PLN.

☐ yes ☐ no

The participant has signed the remuneration receipt.

☐ yes ☐ no

## Adverse Events

Were there any adverse events during this visit? ☐ yes ☐ no

If so, please elaborate:________________________________________________________________________

___________________________________________________________________________________________

___________________________________________________________________________________________

## Continuation of the Study

Is there any reason to discontinue the participation in the study? ☐ yes ☐ no

If so, why: _________________________________________________________________________________

# 6 Online Monitoring (T4-T9)

| **Timepoint** | **Questionnaire** | **Fully answered** | |
| --- | --- | --- | --- |
| T4 |  |  |  |
|  | Daily Hassle (MIMIS) | ☐ yes | ☐ no |
|  | Life events (LEQ) | ☐ yes | ☐ no |
|  | PHQ-ADS | ☐ yes | ☐ no |
|  | Sleep questionnaire | ☐ yes | ☐ no |
|  | Current Mood and Menstrual Cycle | ☐ yes | ☐ no |
| T5 |  |  |  |
|  | Daily Hassle (MIMIS) | ☐ yes | ☐ no |
|  | Life events (LEQ) | ☐ yes | ☐ no |
|  | PHQ-ADS | ☐ yes | ☐ no |
|  | Sleep questionnaire | ☐ yes | ☐ no |
|  | Current Mood and Menstrual Cycle | ☐ yes | ☐ no |
| T6 |  |  |  |
|  | Daily Hassle (MIMIS) | ☐ yes | ☐ no |
|  | Life events (LEQ) | ☐ yes | ☐ no |
|  | PHQ-ADS | ☐ yes | ☐ no |
|  | Sleep questionnaire | ☐ yes | ☐ no |
|  | Current Mood and Menstrual Cycle | ☐ yes | ☐ no |
| T7 |  |  |  |
|  | Daily Hassle (MIMIS) | ☐ yes | ☐ no |
|  | Life events (LEQ) | ☐ yes | ☐ no |
|  | PHQ-ADS | ☐ yes | ☐ no |
|  | Sleep questionnaire | ☐ yes | ☐ no |
|  | Current Mood and Menstrual Cycle | ☐ yes | ☐ no |
| T8 |  |  |  |
|  | Daily Hassle (MIMIS) | ☐ yes | ☐ no |
|  | Life events (LEQ) | ☐ yes | ☐ no |
|  | PHQ-ADS | ☐ yes | ☐ no |
|  | Sleep questionnaire | ☐ yes | ☐ no |
|  | Current Mood and Menstrual Cycle | ☐ yes | ☐ no |
| T9 |  |  |  |
|  | Daily Hassle (MIMIS) | ☐ yes | ☐ no |
|  | Life events (LEQ) | ☐ yes | ☐ no |
|  | PHQ-ADS | ☐ yes | ☐ no |
|  | Sleep questionnaire | ☐ yes | ☐ no |
|  | Current Mood and Menstrual Cycle | ☐ yes | ☐ no |

**Comments Online Monitoring:**

__________________________________________________________________________________________________________________________________________________________________________________________________________________________________________________________________________________________________________________________________________________________________________________________________________________________________________________________________________________________________________________________________________________________________

## Adverse Events

Were there any adverse events during this period? ☐ yes ☐ no

If so, please elaborate:________________________________________________________________________

___________________________________________________________________________________________

___________________________________________________________________________________________

## Continuation of the Study

Is there any reason to discontinue the participation in the study? ☐ yes ☐ no

If so, why: _________________________________________________________________________________

# 7 Study Visit 3 (T9)

## Blood Sample

| **Sampling Date (DD/MM/YY)** |  |
| --- | --- |
| **Sampling Time** |  |
| **Medical technician** |  |
| **Measures Taken *** |  |
| **Comments** |  |

***Amount of blood taken (tubes) and for which purpose.**

## Questionnaire battery

| **No.** | **Questionnaire** | **Fully Answered** | |
| --- | --- | --- | --- |
| 1 | Anxiety Sensitivity Index (ASI) | ☐ yes | ☐ no |
| 3 | Brief Resilience Scale (BRS) | ☐ yes | ☐ no |
| 4 | Cognitive Emotion Regulation Questionnaire (CERQ-short) | ☐ yes | ☐ no |
| 5 | Cognitive Orientation to Problems Experienced questionnaire (Brief COPE) | ☐ yes | ☐ no |
| 6 | Coping Flexibility Scale Revised Questionnaire (CFS-R) | ☐ yes | ☐ no |
| 7 | Context Sensitivity Index (CSI) | ☐ yes | ☐ no |
| 7 | Difficulties in Emotion Regulation Questionnaire (DERS) | ☐ yes | ☐ no |
| 8 | Flexible Emotion Regulation Scale (FlexER-10 – Scale) | ☐ yes | ☐ no |
| 10 | General Self Efficacy Scale (GSE) | ☐ yes | ☐ no |
| 11 | Internal External Locus of Control – 4 (IE-4) | ☐ yes | ☐ no |
| 13 | Life Orientation Test – Revised (LOT-R) | ☐ yes | ☐ no |
| 18 | Oslo 3 Item Social Support Scale (OSSS-3) | ☐ yes | ☐ no |
| 19 | Perceived Positive Appraisal Style Scale; content-focused (PASS-content) | ☐ yes | ☐ no |
| 20 | Perceived Positive Appraisal Style Scale; process-focused (PASS-process) | ☐ yes | ☐ no |
| 21 | Perceived Social Status Scale (PSS-10) | ☐ yes | ☐ no |
| 23 | Revised Symptom Checklist 90 (SCL-90-R)* | ☐ yes | ☐ no |
| 24 | State Trait Anxiety Inventory - trait (STAI-T) | ☐ yes | ☐ no |
| 25 | Psychological Flexibility Questionnaire (PFQ) | ☐ yes | ☐ no |
| 28 | Pittsburgh Sleep Quality Index (PSQI) | ☐ yes | ☐ no |

*If the subjects shows signs of suicidal or self-harming thoughts, they will be re-directed to a page advising them to seek support or help, including the phone number of the local suicide-helpline.

## Adverse Events

Were there any adverse events during this visit? ☐ yes ☐ no

If so, please elaborate:________________________________________________________________________

___________________________________________________________________________________________

___________________________________________________________________________________________

A final discussion of the study has been conducted.

☐ yes ☐ no

All open questions have been clarified.

☐ yes ☐ no

**Comments**:

__________________________________________________________________________________________________________________________________________________________________________________________________________________________________________________________________________________________________________________________________________________________________________________________________________________________________________________________________________________________________________________________________________________________________

________________________________________________________________________________________________________________________________________________________________________________________________________________________________________________________________________________________________________________________________________________________________________________________________________________________________________________________________________________________________________________________________________________________________________________________________________________________________________________________________________________________________________________________________________________________

## Remuneration

The participant has received the remuneration for Visit 3 and the online monitoring 270 Swiss Francs / 240 euro / 750 PLN.

☐ yes ☐ no

The participant has signed the remuneration receipt.

☐ yes ☐ no
