# Supplementary material for: Study protocol: role of the blood-brain barrier in stress resilience: investigating new pathways towards Pharmacological augmentation of stress resilience (a PHASR-PP project study)
Source: BMC Psychol. 2026 Mar 17;14:486. doi: 10.1186/s40359-026-04118-z (PMC13063912; doi:10.1186/s40359-026-04118-z)
Supplement: Supplementary file 3 — Supplementary Material 3: Appendix 3 contains detailed descriptions of the MRI scanning sequences. [file 40359_2026_4118_MOESM3_ESM.docx]

Appendix 1.

**Socio-demographic and anamnestic information**

| **Name** | **Time of assessment** | **Construct and Content** | **Original source** | **German validation** | **Polish validation** |
| --- | --- | --- | --- | --- | --- |
| Anamnesis | - Screening visit; online questionnaire battery (completed on site) | Handedness, general perception of health, history of psychiatric disorders in family, habitual use of drugs (alcohol, cigarettes, cannabis), habitual use of internet, habitual physical activities, habitual activities directed at mental health support (e.g. yoga, meditation, neurofeedback etc.), alcohol/cannabis use frequency, drug abuse status (cocaine, MDMA, ketamine, etc.), ADHD medication use status | self-created (German) | n.a. | self-translated |
| Demographic variables | - online pre-screening questionnaire - Screening visit; online questionnaire battery (completed on site) | Age, sex, language, study status, adoption status, twin status, nationality, country of birth, family ethnicity, housing situation in childhood, siblings status, children status, level of education, occupation, civil status, household and personal income, field of study | self-created (German) | n.a. | self-translated |
| Physical Health (vital signs) | - Screening visit; medical exam | Height, weight, body mass index (BMI), body temperature, blood pressure, heart rate | - | - | - |
| Beck Depression Inventory (BDI) | - online pre-screening questionnaire - Screening visit; medical exam | Symptoms of depression, 21 items | Beck, A. T., Ward, C. H., Mendelson, M., Mock, J., & Erbaugh, J. (1961). An inventory for measuring depression. *Archives of general psychiatry*, *4*(6), 561-571. | Schmitt, M., & Maes, J. (1999). *Vorschlag zur vereinfachung des beck-depressions-inventars (BDI)*. Inst. für Psychologie. | Zawadzki, B., Popiel, A., & Praglowska, E. (2009). Psychometric properties of the polish version of the Aaron T. Beck's depression inventory BDI-II (Charakterystyka psychometryczna polskiej adaptacji Kwestionariusza Depresji BDI-II Aarona T. Becka). *Psychologia-Etologia-Genetyka*, *19*, 71-95. |
| Columbia-Suicide Severity Rating Scale (C-SSRS) | - online pre-screening questionnaire - Screening visit; medical exam | Screening questionnaire for suicidal risk, assessment of suicidal ideation within the past month and lifetime suicidal attempt, 3 items | Posner, K., Brown, G. K., Stanley, B., Brent, D. A., Yershova, K. V., Oquendo, M. A., ... & Mann, J. J. (2011). The Columbia–Suicide Severity Rating Scale: initial validity and internal consistency findings from three multisite studies with adolescents and adults. *American journal of psychiatry*, *168*(12), 1266-1277. | ICON Language Services provides the linguistic validation from the original-language version:  - Translations - the columbia lighthouse project (2025) The Columbia Lighthouse Project - Home of the Columbia-Suicide Severity Rating Scale (C-SSRS): a series of simple, plain-language questions that anyone can use to assess suicide risk. Available at: https://cssrs.columbia.edu/the-columbia-scale-c-ssrs/translations/#lv (Accessed: 30 April 2025).  - Integrating AI into clinical research (no date) ICON plc \| Clinical Research Organisation (CRO) for Drug Development. Available at: https://www.iconplc.com/ (Accessed: 30 April 2025). | Mokros, Ł., Rawska-Kabacińska, A., Świtaj, P., Wieczorek, Ł., Jabłońska, A., & Anczewska, M. (2024). Adaptation and validation of the Columbia-Suicide Severity Rating Scale (C-SSRS)-screen version. *Psychiatria Polska*. |
| General Health Questionnaire (GHQ-28) | - online pre-screening questionnaire | Symptoms of anxiety, depression, insomnia, social problems as well as somatic symptoms. This inventory is designed to capture the inability to carry out normal functions and the appearance of new and distressing phenomena in the general population, 28 items | Goldberg, D. P., Gater, R., Sartorius, N., Ustun, T. B., Piccinelli, M., Gureje, O., & Rutter, C. (1997). The validity of two versions of the GHQ in the WHO study of mental illness in general health care. *Psychological medicine*, *27*(1), 191-197. | Hobi, V., Gerhard, U., & Gutzwiller, F. (1989). A report on experiences using Goldberg's GHQ (General Health Questionnaire). *Schweizerische Rundschau fur Medizin Praxis= Revue Suisse de Medecine Praxis*, *78*(9), 219-225. | Makowska, Z., Merecz, D., Moscicka, A., & Kolasa, W. (2002). The Validity Of General Health Questionnaires, GHQ-12 and GHQ-28, in Mental Health Studies of Working People. *International Journal of Occupational Medicine & Environmental Health*, *15*(4). |
| Life Events Questionnaire (LEQ) | - online pre-screening questionnaire | 28 stressful life events (e.g., death of a friend or family member, separation or divorce of the parents, illness or injury). For each event, participants indicate whether and at what age it has occurred and how positive or burdensome it has been experienced, 28 items | Canli, T., Qiu, M., Omura, K., Congdon, E., Haas, B. W., Amin, Z., ... & Lesch, K. P. (2006). Neural correlates of epigenesis. *Proceedings of the National Academy of Sciences*, *103*(43), 16033-16038. | Chmitorz, A., Neumann, R. J., Kollmann, B., Ahrens, K. F., Öhlschläger, S., Goldbach, N., ... & Reif, A. (2021). Longitudinal determination of resilience in humans to identify mechanisms of resilience to modern-life stressors: the longitudinal resilience assessment (LORA) study. *European Archives of Psychiatry and Clinical Neuroscience*, *271*, 1035-1051. | self-translated, not validated |
| Mini Mental State Examination (MMSE) | - Screening visit; medical exam | Structured test measuring individuals cognitive functioning, used to screen for cognitive impairment. Covers basic mental functions, such as: orientation, memory (encoding and recall), attention, visuo-spatial skills, 30 items | Folstein, M. F., Folstein, S. E., & McHugh, P. R. (1975). “Mini-mental state”: a practical method for grading the cognitive state of patients for the clinician. *Journal of psychiatric research*, *12*(3), 189-198. | Beyermann, S., Trippe, R. H., Bähr, A. A., & Püllen, R. (2013). Mini-mental-status-test im stationären geriatrischen Bereich: eine evaluation der diagnostischen Qualität. *Zeitschrift für Gerontologie und Geriatrie*, *46*(8). | Stańczak, J. (2013). *MMSE: polska normalizacja*. Pracownia Testów Psychologicznych Polskiego Towarzystwa Psychologicznego. |
