# Supplementary material for: Study protocol: role of the blood-brain barrier in stress resilience: investigating new pathways towards Pharmacological augmentation of stress resilience (a PHASR-PP project study)
Source: BMC Psychol. 2026 Mar 17;14:486. doi: 10.1186/s40359-026-04118-z (PMC13063912; doi:10.1186/s40359-026-04118-z)
Supplement: Supplementary file 4 — Supplementary Material 4: Appendix 4 contains the Case Report Form (CRF) [file 40359_2026_4118_MOESM4_ESM.docx]

Appendix 2.

**Psycho-social questionnaires**

| **Questionnaire** | **Time of assessment** | **Construct and Content** | **Original source** | **German validation** | **Polish validation** |
| --- | --- | --- | --- | --- | --- |
| Anxiety Sensitivity Index (ASI-3) | - T0 online questionnaire battery (completed on site) - T3 online questionnaire battery (completed on site) - T9 online questionnaire battery (completed on site) | Measure of anxiety sensitivity, especially in domains of physical concerns (e.g. fear of a rapid heart beat), cognitive concerns (e.g. fear of cognitive dyscontrol), and social concerns (e.g. fear of observable anxiety symptoms in social settings), 18 items | Olthuis, J. V., Watt, M. C., & Stewart, S. H. (2014). Anxiety Sensitivity Index (ASI-3) subscales predict unique variance in anxiety and depressive symptoms. *Journal of Anxiety Disorders*, *28*(2), 115-124. | Kemper, C. J., Ziegler, M., & Taylor, S. (2009). Überprüfung der psychometrischen Qualität der deutschen Version des Angstsensitivitätsindex-3. *Diagnostica*, *55*(4), 223-233. | Michałowski, J. M., Holas, P., & Zvolensky, M. J. (2014). Polish adaptation and psychometric validation of the anxiety sensitivity index-III. *Journal of individual differences*. |
| Brief Resilience Scale (BRS) | - T0 online questionnaire battery (completed on site) - T3 online questionnaire battery (completed on site) - T9 online questionnaire battery (completed on site) | The subjective ability to cope with and recover from stress, 10 items | Smith, B. W., Dalen, J., Wiggins, K., Tooley, E., Christopher, P., & Bernard, J. (2008). The brief resilience scale: assessing the ability to bounce back. *International journal of behavioral medicine*, *15*, 194-200. | Chmitorz, A., Wenzel, M., Stieglitz, R. D., Kunzler, A., Bagusat, C., Helmreich, I., ... & Tüscher, O. (2018). Population-based validation of a German version of the Brief Resilience Scale. *PloS one*, *13*(2), e0192761. | Konaszewski, K., Niesiobędzka, M., & Surzykiewicz, J. (2020). Validation of the Polish version of the Brief Resilience Scale (BRS). *PloS one*, *15*(8), e0237038. |
| Childhood Trauma Questionnaire (CTQ) | - T3 online questionnaire battery (completed on site) | Measure of individual’s experiences of child abuse and neglect (emotional, physical, and sexual abuse, and emotional and physical neglect), 29 items | Bernstein, D. P., Stein, J. A., Newcomb, M. D., Walker, E., Pogge, D., Ahluvalia, T., ... & Zule, W. (2003). Development and validation of a brief screening version of the Childhood Trauma Questionnaire. *Child abuse & neglect*, *27*(2), 169-190. | Dudeck, M., Vasic, N., Otte, S., Streb, J., Wingenfeld, K., Grabe, H. J., ... & Spitzer, C. (2015). Factorial validity of the short form of the childhood trauma questionnaire (CTQ—SF) in German psychiatric patients, inmates, and university students. *Psychological reports*, *116*(3), 685-703. | self-translated, not validated |
| Cognitive emotion regulation questionnaire (CERQ short form) | - T0 online questionnaire battery (completed on site) - T3 online questionnaire battery (completed on site) - T9 online questionnaire battery (completed on site) | Measure to assess individuals tendencies of using nine different cognitive emotion regulation strategies (e.g. rumination, catastrophizing, positive reappraisal, acceptance) in light of stressful life events. Shortened version of full 36-item scale to 20 items (2 items per strategy) | Garnefski, N., & Kraaij, V. (2006). Cognitive emotion regulation questionnaire – development of a short 18-item version (CERQ-short). *Personality and individual differences*, *41*(6), 1045-1053. | Loch, N., Hiller, W., & Witthöft, M. (2011). Der cognitive emotion regulation questionnaire (CERQ). *Zeitschrift für klinische psychologie und psychotherapie*. | Marszał-Wiśniewska, M., & Fajkowska, M. (2010). Właściwości psychometryczne Kwestionariusza Poznawczej Regulacji Emocji (Cognitive Emotion Regulation Questionnaire; CERQ)-wyniki badań na polskiej próbie. *Studia Psychologiczne (eng. Psychological Studies)*, *49*. |
| Coping Flexibility Scale - Revised (CFS-R) | - T0 online questionnaire battery (completed on site) - T3 online questionnaire battery (completed on site) - T9 online questionnaire battery (completed on site) | Measure of one’s ability to modify his/her coping strategies adaptively to meet the demands of different stressful situations, assessed with two subscales: Evaluation Coping Scale and Adaptive Coping Scale, 12 items | Kato, T. (2020). Examination of the coping flexibility hypothesis using the coping flexibility scale-revised. *Frontiers in Psychology*, *11*, 561731. | self-translated, not validated | self-translated, not validated |
| Coping Orientation to Problems Experienced (Brief COPE) | - T0 online questionnaire battery (completed on site) - T3 online questionnaire battery (completed on site) - T9 online questionnaire battery (completed on site) | Emotion regulation strategies such as self-distraction, active coping, denial, substance use, use of emotional support, use of instrumental support, behavioral disengagement, venting, positive reframing, planning, humor, acceptance, religion, and self-blame, 28 items | Carver, C. S. (1997). You want to measure coping but your protocol’s too long: Consider the brief cope. *International journal of behavioral medicine*, *4*(1), 92-100. | Hanfstingl, B., Gnambs, T., Fazekas, C., Gölly, K. I., Matzer, F., & Tikvić, M. (2023). The Dimensionality of the Brief COPE before and during the COVID-19 Pandemic. *Assessment*, *30*(2), 287-301. | Ogińska-Bulik, N., & Juczyński, Z. (2009). NPSR–Narzędzia Pomiaru Stresu i Radzenia Sobie ze Stresem. *Pracownia Testów Psychologicznych*. |
| Context Sensitivity Index (CSI) | - T0 online questionnaire battery (completed on site) - T3 online questionnaire battery (completed on site) - T9 online questionnaire battery (completed on site) | Scenario-based index of context sensitivity measuring one’s ability to perceive and respond flexibly to contextual cues, especially in stressful or emotionally charged situations, 6 scenarios with 3 items for each scenario | Bonanno, G. A., Maccallum, F., Malgaroli, M., & Hou, W. K. (2020). The Context Sensitivity Index (CSI): Measuring the ability to identify the presence and absence of stressor context cues. *Assessment*, *27*(2), 261-273. | self-translated, not validated | self-translated, not validated |
| Difficulties in Emotion Regulation Questionnaire (DERS) | - T0 online questionnaire battery (completed on site) - T3 online questionnaire battery (completed on site) - T9 online questionnaire battery (completed on site) | Measure of emotional dysregulation. Higher scores indicate problems in four dimensions of emotion regulation, i.e. awareness and understanding of emotions, acceptance of emotions, ability to engage in goal-directed behavior, and refrain from impulsive behavior, when experiencing negative emotions, and access to emotion regulation strategies perceived as effective, 36 items | Gratz, K. L., & Roemer, L. (2004). Multidimensional assessment of emotion regulation and dysregulation: Development, factor structure, and initial validation of the difficulties in emotion regulation scale. *Journal of psychopathology and behavioral assessment*, *26*, 41-54. | Gutzweiler, R., & In-Albon, T. (2019). Überprüfung der Gütekriterien der deutschen Version der Difficulties in Emotion Regulation Scale in einer klinischen und einer Schülerstichprobe Jugendlicher. *Zeitschrift für klinische Psychologie und Psychotherapie*. | self-translated, not validated |
| Flexible Emotion Regulation Questionnaire (FlexER-Scale) | - T0 online questionnaire battery (completed on site) - T3 online questionnaire battery (completed on site) - T9 online questionnaire battery (completed on site) | Measure of emotion regulation flexibility, capturing the adaptive, context- and goal-dependent implementation of variable ER strategies at the trait level, 10 items | Gärtner, A., Scheffel, C., Schweikert, T., & Dörfel, D. (2025). The FlexER-Scale: A new Self-report Measure of Individual Differences in Emotion Regulation Flexibility. | Gärtner, A., Scheffel, C., Schweikert, T., & Dörfel, D. (2025). The FlexER-Scale: A new Self-report Measure of Individual Differences in Emotion Regulation Flexibility. | self-translated, not validated |
| General Self-Efficacy Scale (GSE) | - T0 online questionnaire battery (completed on site) - T3 online questionnaire battery (completed on site) - T9 online questionnaire battery (completed on site) | Measure of one's belief in his/her competence to tackle novel tasks and cope with adversity in a broad range of stressful or challenging encounters, 10 items | Schwarzer, R., & Jerusalem, M. (1995). Generalized self-efficacy scale. *J. Weinman, S. Wright, & M. Johnston, Measures in health psychology: A user’s portfolio. Causal and control beliefs*, *35*(37), 82-003. | Luszczynska, A., Scholz, U., & Schwarzer, R. (2005). The general self-efficacy scale: multicultural validation studies. *The Journal of psychology*, *139*(5), 439-457. | Luszczynska, A., Scholz, U., & Schwarzer, R. (2005). The general self-efficacy scale: multicultural validation studies. *The Journal of psychology*, *139*(5), 439-457. |
| Internal External Locus of Control (IE-4) | - T0 online questionnaire battery (completed on site) - T3 online questionnaire battery (completed on site) - T9 online questionnaire battery (completed on site) | Measure of one’s belief about whether outcomes of their behavior are determined by one’s actions or by forces outside one’s control, 4 items | Kovaleva, A. (2012). *The IE-4: Construction and validation of a short scale for the assessment of locus of control* (Vol. 9, p. 130). DEU. | Kovaleva, A. (2012). *The IE-4: Construction and validation of a short scale for the assessment of locus of control* (Vol. 9, p. 130). DEU. | self-translated, not validated |
| Life Events Questionnaire (LEQ) | - Monthly monitoring battery (completed online) | 28 stressful life events (e.g., death of a friend or family member, separation or divorce of the parents, illness or injury). For each event, participants indicate whether and at what age it has occurred and how positive or burdensome it has been experienced, 28 items | Canli, T., Qiu, M., Omura, K., Congdon, E., Haas, B. W., Amin, Z., ... & Lesch, K. P. (2006). Neural correlates of epigenesis. *Proceedings of the National Academy of Sciences*, *103*(43), 16033-16038. | Chmitorz, A., Neumann, R. J., Kollmann, B., Ahrens, K. F., Öhlschläger, S., Goldbach, N., ... & Reif, A. (2021). Longitudinal determination of resilience in humans to identify mechanisms of resilience to modern-life stressors: the longitudinal resilience assessment (LORA) study. *European Archives of Psychiatry and Clinical Neuroscience*, *271*, 1035-1051. | self-translated, not validated |
| Life Orientation Test (LOT-R) | - T0 online questionnaire battery (completed on site) - T3 online questionnaire battery (completed on site) - T9 online questionnaire battery (completed on site) | Dispositional optimism and pessimism, 10 items | Chiesi, F., Galli, S., Primi, C., Innocenti Borgi, P., & Bonacchi, A. (2013). The Accuracy of the Life Orientation Test–Revised (LOT–R) in measuring dispositional optimism: Evidence from item response theory analyses. *Journal of personality assessment*, *95*(5), 523-529. | Glaesmer, H., Rief, W., Martin, A., Mewes, R., Brähler, E., Zenger, M., & Hinz, A. (2012). Psychometric properties and population‐based norms of the Life Orientation Test Revised (LOT‐R). *British journal of health psychology*, *17*(2), 432-445. | Juczyński, Z. (2001). *Narzędzia pomiaru w promocji i psychologii zdrowia* (Vol. 188). Warszawa: Pracownia Testów Psychologicznych Polskiego Towarzystwa Psychologicznego. |
| Mainz Inventory of Microstressors (MIMIS) | - Monthly monitoring battery (completed online) | Inventory of objective stressors that individuals might have experienced during the course of one week, with the assessment of perceived severity of each stressor, 58 items | Chmitorz, A., Kurth, K., Mey, L. K., Wenzel, M., Lieb, K., Tüscher, O., ... & Kalisch, R. (2020). Assessment of microstressors in adults: questionnaire development and ecological validation of the Mainz inventory of microstressors. *JMIR Mental Health*, *7*(2), e14566. | Chmitorz, A., Kurth, K., Mey, L. K., Wenzel, M., Lieb, K., Tüscher, O., ... & Kalisch, R. (2020). Assessment of microstressors in adults: questionnaire development and ecological validation of the Mainz inventory of microstressors. *JMIR Mental Health*, *7*(2), e14566. | self-translated, not validated |
| Maltreatment and Abuse Chronology of Exposure (MACE) | - T0 online questionnaire battery (completed on site) | Measure of exposure to traumatic events, such as abuse and maltreatment up to the age of 18, with the option of noting at what age the events took place, 52 items | Teicher, M. H., & Parigger, A. (2015). The ‘Maltreatment and Abuse Chronology of Exposure’ (MACE) scale for the retrospective assessment of abuse and neglect during development. *PLoS one*, *10*(2), e0117423. | Isele, D., Teicher, M. H., Ruf-Leuschner, M., Elbert, T., Kolassa, I. T., Schury, K., & Schauer, M. (2014). KERF–ein Instrument zur umfassenden Ermittlung belastender Kindheitserfahrungen. *Zeitschrift für Klinische Psychologie und Psychotherapie*. | Chęć, M., Michałowska, S., Rachubińska, K., Konieczny, K., & Samochowiec, A. (2025). Polish adaptation of ‘Maltreatment and Abuse Chronology of Exposure’scale. *PloS one*, *20*(4), e0321046. |
| NEO - Five Factor Inventory (NEO-FFI) - agreeableness & neuroticism subscales | - T0 online questionnaire battery (completed on site) | Assessment of the Big Five personality traits, for purposes of this study we only used Agreeableness and Neuroticism subscales, each containing 12 items | Körner, A., Czajkowska, Z., Albani, C., Drapeau, M., Geyer, M., & Braehler, E. (2015). Efficient and valid assessment of personality traits: population norms of a brief version of the NEO Five-Factor Inventory (NEO-FFI). *Archives of Psychiatry & Psychotherapy*, *17*(1). | Körner, A., Drapeau, M., Albani, C., Geyer, M., Schmutzer, G., & Brähler, E. (2008). Deutsche normierung des NEO-Fünf-Faktoren-Inventars (NEO-FFI) German norms for the NEO-five factor inventory. *Zeitschrift für Medizinische Psychologie*, *17*(2-3), 133-144. | Zawadzki, B., Strelau, J., Szczepaniak, P., & Śliwińska, M. (1998). Inwentarz osobowości NEO-FFI Costy i McCrae. Adaptacja polska. Podręcznik. *Pracownia Testów Psychologicznych PTP*. |
| Oslo 3 Item Social Support Scale (OSSS-3) | - T0 online questionnaire battery (completed on site) - T3 online questionnaire battery (completed on site) - T9 online questionnaire battery (completed on site) | Measure of individuals social support - extent to which one experiences or expects obtaining support from family, friends and neighbors if required, and the size of one’s social network, as well as the degree of mutual interest within it, 3 items | Kocalevent, R. D., Berg, L., Beutel, M. E., Hinz, A., Zenger, M., Härter, M., ... & Brähler, E. (2018). Social support in the general population: standardization of the Oslo social support scale (OSSS-3). *BMC psychology*, *6*, 1-8. | Kocalevent, R. D., Berg, L., Beutel, M. E., Hinz, A., Zenger, M., Härter, M., ... & Brähler, E. (2018). Social support in the general population: standardization of the Oslo social support scale (OSSS-3). *BMC psychology*, *6*, 1-8. | self-translated, not validated |
| Perceived Positive Appraisal Style Scale – content-focused (PASS-content) | - T0 online questionnaire battery (completed on site) - T3 online questionnaire battery (completed on site) - T9 online questionnaire battery (completed on site) | Assessment of the perceived tendency to generate positive appraisals in challenging situations, 14 items | Petri-Romão, P., Engen, H., Rupanova, A., Puhlmann, L., Zerban, M., Neumann, R. J., ... & Kalisch, R. (2024). Self-report assessment of Positive Appraisal Style (PAS): development of a process-focused and a content-focused questionnaire for use in mental health and resilience research. *Plos one*, *19*(2), e0295562. | Petri-Romão, P., Engen, H., Rupanova, A., Puhlmann, L., Zerban, M., Neumann, R. J., ... & Kalisch, R. (2024). Self-report assessment of Positive Appraisal Style (PAS): development of a process-focused and a content-focused questionnaire for use in mental health and resilience research. *Plos one*, *19*(2), e0295562. | self-translated, not validated |
| Perceived Positive Appraisal Style Scale – process-focused (PASS-process) | - T0 online questionnaire battery (completed on site) - T3 online questionnaire battery (completed on site) - T9 online questionnaire battery (completed on site) | Assessment of the perceived tendency to employ positive appraisal processes in challenging situations, 10 items | Petri-Romão, P., Engen, H., Rupanova, A., Puhlmann, L., Zerban, M., Neumann, R. J., ... & Kalisch, R. (2024). Self-report assessment of Positive Appraisal Style (PAS): development of a process-focused and a content-focused questionnaire for use in mental health and resilience research. *Plos one*, *19*(2), e0295562. | Petri-Romão, P., Engen, H., Rupanova, A., Puhlmann, L., Zerban, M., Neumann, R. J., ... & Kalisch, R. (2024). Self-report assessment of Positive Appraisal Style (PAS): development of a process-focused and a content-focused questionnaire for use in mental health and resilience research. *Plos one*, *19*(2), e0295562. | self-translated, not validated |
| Perceived Stress Scale (PSS-10) | - T0 online questionnaire battery (completed on site) - T3 online questionnaire battery (completed on site) - T9 online questionnaire battery (completed on site) | Degree to which participants appraise situations in their lives as stressful, unpredictable, uncontrollable, and overloaded, 10 items | Roberti, J. W., Harrington, L. N., & Storch, E. A. (2006). Further psychometric support for the 10‐item version of the perceived stress scale. *Journal of College Counseling*, *9*(2), 135-147. | Klein, E. M., Brähler, E., Dreier, M., Reinecke, L., Müller, K. W., Schmutzer, G., ... & Beutel, M. E. (2016). The German version of the Perceived Stress Scale–psychometric characteristics in a representative German community sample. *BMC psychiatry*, *16*, 1-10. | Ogińska-Bulik, N., & Juczyński, Z. (2009). NPSR–Narzędzia Pomiaru Stresu i Radzenia Sobie ze Stresem. *Pracownia Testów Psychologicznych*. |
| Patient Health Questionnaire - Anxiety and Depression Scale (PHQ-ADS) | - Monthly monitoring battery (completed online) | Composite measure of psychological distress. Consists of two scales - PHQ-9 and GAD-7.  Patient Health Questionnaire-9 (PHQ-9) assesses the prevalence of depressive symptoms (e.g. anhedonia, hopelessness, lack of sleep, poor appetite) within the past two weeks, 9 items.  Generalised Anxiety Disorder Scale (GAD-7) assesses symptoms of anxiety (e.g. excessive worrying, restlessness, trouble relaxing) within the past two weeks, 7 items | Kroenke, K., Wu, J., Yu, Z., Bair, M. J., Kean, J., Stump, T., & Monahan, P. O. (2016). Patient health questionnaire anxiety and depression scale: initial validation in three clinical trials. *Psychosomatic medicine*, *78*(6), 716-727. | GAD-7  Kliem, S., Sachser, C., Lohmann, A., Baier, D., Brähler, E., Fegert, J. M., & Gündel, H. (2025). Psychometric evaluation and community norms of the GAD-7, based on a representative German sample. *Frontiers in Psychology*, *16*, 1526181.  PHQ-9  Kliem, S., Sachser, C., Lohmann, A., Baier, D., Brähler, E., Gündel, H., & Fegert, J. M. (2024). Psychometric evaluation and community norms of the PHQ-9, based on a representative German sample. *Frontiers in Psychiatry*, *15*, 1483782. | GAD-7  Basińska, B. A., & Kwissa-Gajewska, Z. (2023). Psychometric Properties of the Polish Version of the Generalized Anxiety Disorder 7-Item Scale (GAD-7) in a Non-Clinical Sample of Employees during Pandemic Crisis. *International Journal of Occupational Medicine and Environmental Health*, *36*(4), 493.  PHQ-9  Ślusarska, B. J., Nowicki, G., Piasecka, H., Zarzycka, D., Mazur, A., Saran, T., & Bednarek, A. (2019). Validation of the Polish language version of the Patient Health Questionnaire-9 in a population of adults aged 35–64. *Annals of Agricultural and Environmental Medicine*, *26*(3), 420-424. |
| Pittsburgh Sleep Quality Index (PSQI) | - T0 online questionnaire battery (completed on site) - T3 online questionnaire battery (completed on site) - T9 online questionnaire battery (completed on site) | Measure of subjective sleep quality, duration and disturbances, 17 items grouped into 7 components | Buysse, D. J., Reynolds III, C. F., Monk, T. H., Berman, S. R., & Kupfer, D. J. (1989). The Pittsburgh Sleep Quality Index: a new instrument for psychiatric practice and research. *Psychiatry research*, *28*(2), 193-213. | Hinz, A., Glaesmer, H., Brähler, E., Löffler, M., Engel, C., Enzenbach, C., ... & Sander, C. (2017). Sleep quality in the general population: psychometric properties of the Pittsburgh Sleep Quality Index, derived from a German community sample of 9284 people. *Sleep medicine*, *30*, 57-63. | Badzio-Jagiełło, H., Nowicki, Z., Jakitowicz, J., Majkowicz, M. (1999). Kwestionariusz zaburzeń snu u pacjentów z zaburzeniami psychicznymi – ocena psychometryczna. W: Z. Nowicki, W. Szelenberger (red.), Zaburzenia snu. Diagnostyka i leczenie – wybrane zagadnienia. Kraków: Biblioteka Psychiatrii Polskiej. |
| Psychological Flexibility Questionnaire (PFQ) | - T0 online questionnaire battery (completed on site) - T3 online questionnaire battery (completed on site) - T9 online questionnaire battery (completed on site) | Subjective psychological flexibility, assessed via five factors including positive perception of change, characterization of the self as flexible, self-characterization as open and innovative, a perception of reality as dynamic and changing, and a perception of reality as multifaceted, 20 items | Ben-Itzhak, S., Bluvstein, I., & Maor, M. (2014). The psychological flexibility questionnaire (PFQ): Development, reliability and validity. | self-translated, not validated | self-translated, not validated |
| Revised Symptom Checklist 90 (SCL-90-R) | - T0 online questionnaire battery (completed on site) - T3 online questionnaire battery (completed on site) - T9 online questionnaire battery (completed on site) | Psychological distress in terms of nine primary symptom dimensions including somatization, obsessive–compulsive, interpersonal sensitivity, depression, anxiety, hostility, phobic anxiety, paranoid ideation, and psychoticism, 90 items | Derogatis, L. R., & Cleary, P. A. (1977). Confirmation of the dimensional structure of the SCL‐90: A study in construct validation. *Journal of clinical psychology*, *33*(4), 981-989. | Schmitz, N., Hartkamp, N., Kiuse, J., Franke, G. H., Reister, G., & Tress, W. (2000). The symptom check-list-90-R (SCL-90-R): a German validation study. *Quality of Life Research*, *9*, 185-193. | self-translated, not validated |
| Sleep questionnaire  Sleep Regularity Questionnaire (SRQ) + 2 items from Pittsburgh Sleep Quality Index (PSQI) | - Monthly monitoring battery (completed online) | SRQ is a measure to assess how consistent a person's sleep-wake patterns (e.g. regularity in wake-up and to-bed times, sleep duration etc.) are over time. In the case of the current study we measure the sleep regularity pattern over the course of the past two weeks in the online monitoring assessment, 10 items  From PSQI we measure the average sleep duration and quality in the past two weeks, 2 items | Dzierzewski, J. M., Donovan, E. K., & Sabet, S. M. (2021). The sleep regularity questionnaire: development and initial validation. *Sleep medicine*, *85*, 45-53. | self-translated, not validated | self-translated, not validated |
| State trait anxiety inventory (trait) (STAI-T) | - T0 online questionnaire battery (completed on site) - T3 online questionnaire battery (completed on site) - T9 online questionnaire battery (completed on site) | Symptoms of anxiety as a general trait, 20 items | Spielberger, C. D., Gonzalez-Reigosa, F., Martinez-Urrutia, A., Natalicio, L. F., & Natalicio, D. S. (1971). The state-trait anxiety inventory. *Revista Interamericana de Psicologia/Interamerican journal of psychology*, *5*(3 & 4). | Laux, L. (1981). Das State-Trait-Angstinventar (STAI): theoretische grundlagen und handanweisung. | Spielberger, C. D., Strelau, J., Tysarczyk, M., & Wrześniewski, K. (2006). Polska adaptacja STAI [The Polish adaptation of the STAI]. Polska adaptacja STAI. Inwentarz Stanu i Cechy Lęku [The Polish adaptation of the STAI. State Trait Anxiety Inventory], 3-17. |
| Vividness of Visual Imagery Questionnaire (VVIQ) | - T0 online questionnaire battery (completed on site) | Measure of mental, visual imagery vividness. It consists of 16 items, grouped into 4 scenarios (e.g. imagining a picture of a close relative, or a rising sun) for which individuals are asked to create a detailed picture and rate the vividness of particular details | Marks, D. F. (1973). Vividness of visual imagery questionnaire. *Journal of Mental Imagery*. | Jungmann, S. M., Becker, F., & Witthöft, M. (2022). Erfassung der Lebendigkeit mentaler Vorstellungsbilder: Adaptation und Validierung deutschsprachiger Versionen des Vividness of Visual Imagery Questionnaire (VVIQ) und des Plymouth Sensory Imagery Questionnaire (PSI-Q) [Measuring the vividness of mental images: An adaptation and validation of German versions of the Vividness of Visual Imagery Questionnaire (VVIQ) and the Plymouth Sensory Imagery Questionnaire (PSI-Q)]. *Diagnostica, 68*(3), 125–136. | Jankowska, D. M., & Karwowski, M. (2020). *Visual imagery and creativity: The Polish version of vividness of visual imagery questionnaire and its links with creativity*. |
| Mood and Menstrual Cycle Status | - Monthly monitoring battery (completed online) | Questions on the current mood and menstrual cycle status of the participant at the moment of completing the monthly monitoring battery | Self Created (English) | Self-translated | Self-translated |
