# Supplementary material for: Study protocol: role of the blood-brain barrier in stress resilience: investigating new pathways towards Pharmacological augmentation of stress resilience (a PHASR-PP project study)
Source: BMC Psychol. 2026 Mar 17;14:486. doi: 10.1186/s40359-026-04118-z (PMC13063912; doi:10.1186/s40359-026-04118-z)
Supplement: Supplementary file 5 — Supplementary Material 5: Appendix 5 contains the SPIRIT 2025 checklist [50] [file 40359_2026_4118_MOESM5_ESM.docx]

Appendix 3.

| **Sequence** | **TR (ms)** | **TE (ms)** | **Flip angle (°)** | **FOV (mm)** | **Voxel size (mm)** | **Total acquisition time (min)** | **Additional parameters** |
| --- | --- | --- | --- | --- | --- | --- | --- |
| T1-weighted MPRAGE | 2500 | 2.22 | 8 | 256 | 0.8 × 0.8 × 0.8 | 5 | - |
| Multi-TE pseudo-continuous (ME-pcASL) | 4500 | [17.30, 51.90, 86.50, 121.10, 155.70, 190.30, 224.90, 259.50] | 120 | 320 | 2.5 × 2.5 × 5.0 | 8 | - matrix size = 64 × 128 × 32 - FOV phase = 50% - PLD=600ms - sub-bolus duration = 1000ms - Hadamard-4 matrix - turbo factor = 2 - EPI factor = 16 - bandwidth = 2440 Hz/Px - GRAPPA = 2 - 3D GRASE readout |
| Multi-echo multi-band EPI sequences for rsfMRI | 1435 | [12, 28.24, 44.48, 60.72] | 65 | 215 | 2.5 × 2.5 × 2.5 | 10 | - multi-band factor = 3 - sequence adopted from the Human Connectome Project from the Center for Magnetic Resonance Research, University of Minnesota |
| T2-weighted FLAIR | 9000 | 83 | 150 | 220 | 0.7 × 0.7 × 3.0 | 5 | - |
| Diffusion prepared pseudo-continuous (DP-pcASL) sequence | 4100 | 31.1 | 120 | 240 | 2.5 × 2.5 × 3.0 | 8 | - matrix size = 96 × 96 x 40 - PLD = 500/1000/1500/2000/25000 ms - label/control duration = 1500 ms - turbo factor = 14 - EPI factor = 64 - bandwidth = 2604 Hz/Px - 3D GRASE readout |

**Neuroimaging measures (in order of scan acquisition)**
